# Improving photosensitization for photochemical CO_2_-to-CO conversion

**DOI:** 10.1093/nsr/nwaa112

**Published:** 2020-05-28

**Authors:** Ping Wang, Ru Dong, Song Guo, Jianzhang Zhao, Zhi-Ming Zhang, Tong-Bu Lu

**Affiliations:** MOE International Joint Laboratory of Materials Microstructure, Institute for New Energy Materials and Low Carbon Technologies, School of Materials Science and Engineering, Tianjin University of Technology, Tianjin 300384, China; MOE International Joint Laboratory of Materials Microstructure, Institute for New Energy Materials and Low Carbon Technologies, School of Materials Science and Engineering, Tianjin University of Technology, Tianjin 300384, China; MOE International Joint Laboratory of Materials Microstructure, Institute for New Energy Materials and Low Carbon Technologies, School of Materials Science and Engineering, Tianjin University of Technology, Tianjin 300384, China; State Key Laboratory of Fine Chemicals, School of Chemical Engineering, Dalian University of Technology, Dalian 116024, China; MOE International Joint Laboratory of Materials Microstructure, Institute for New Energy Materials and Low Carbon Technologies, School of Materials Science and Engineering, Tianjin University of Technology, Tianjin 300384, China; MOE International Joint Laboratory of Materials Microstructure, Institute for New Energy Materials and Low Carbon Technologies, School of Materials Science and Engineering, Tianjin University of Technology, Tianjin 300384, China

**Keywords:** photosensitization, photocatalysis, CO_2_ reduction, excited state, Ru(II) complexes

## Abstract

Inspired by nature, improving photosensitization represents a vital direction for the development of artificial photosynthesis. The sensitization ability of photosensitizers (PSs) reflects in their electron-transfer ability, which highly depends on their excited-state lifetime and redox potential. Herein, for the first time, we put forward a facile strategy to improve sensitizing ability via finely tuning the excited state of Ru(II)-PSs (**Ru-1**–**Ru-4**) for efficient CO_2_ reduction. Remarkably, [Ru(Phen)_2_(3-pyrenylPhen)]^2+^ (**Ru-3**) exhibits the best sensitizing ability among **Ru-1**–**Ru-4**, over 17 times higher than that of typical Ru(Phen)_3_^2+^. It can efficiently sensitize a dinuclear cobalt catalyst for CO_2_-to-CO conversion with a maximum turnover number of 66 480. Systematic investigations demonstrate that its long-lived excited state and suitable redox driving force greatly contributed to this superior sensitizing ability. This work provides a new insight into dramatically boosting photocatalytic CO_2_ reduction via improving photosensitization.

## INTRODUCTION

**Figure 1.**
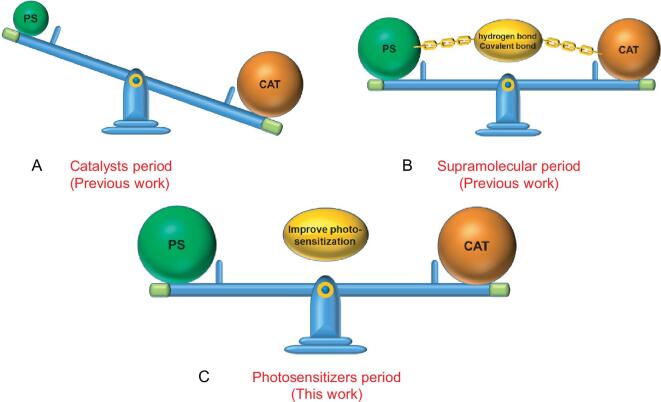
Evolution process of the photocatalytic system for CO_2_ reduction. (A) The study was mainly focused on the development of highly active catalysts (CATs) during this period. (B) Non-covalent/covalent supramolecular assembly has been developed over the past decades. (C) In this work, we open a new way to boost photocatalytic CO_2_ reduction by improving the photosensitization ability of photosensitizers (PSs).

Solar-driven reduction of CO_2_ into energy-rich fuels, such as CO, HCOOH and CH_3_OH, has been conceived of as a highly promising approach to solve the energy crisis and environmental pollution [1–6]. In the past decades, great efforts have long been devoted to improving the photocatalytic activity and selectivity for efficient CO_2_ conversion. Throughout the molecular photocatalytic systems, numerous catalysts, such as complexes of Re, Ru, Fe, Co and Ni, have been developed [7–13] with detailed study of their catalytic mechanism [14–17]. In light of their being relatively comprehensively studied, more and more attention has begun to shift to accelerate electron transfer between catalyst and antenna molecules to promote CO_2_ reduction. For example, Ishitani and several groups have explored efficient photocatalytic systems through combining photosensitizers (PSs) with catalysts via covalent bonds [8,9,18–20]. These systems exhibited enhanced catalytic ability compared to that of separated systems owing to their more efficient electron transfer via intramolecular process. However, this strategy was limited by the complex synthesis and lack of flexibility in investigating different PSs and catalysts. Very recently, Cheung *et al.* discovered that the hydrogen-bonding interaction between PSs and catalysts can improve the catalytic performance [21]. Nevertheless, these H-bonds can be easily disrupted by external factors, e.g. temperature and the solvents of N,N-Dimethylformamide (DMF) and H_2_O. Accordingly, it is highly necessary yet remains greatly challenging to develop an alternative strategy for dramatically boosting photocatalytic CO_2_ reduction (Fig. 1).

PSs, as the light-harvesting main body, have been widely used as a key mediator for efficient electron transfer between catalysts and electron donors in both natural and artificial photosynthetic systems [3,9,22–24]. Currently, improving the photosensitization ability of PSs for enhancing photocatalytic performance for CO_2_ reduction is still in its infancy [24,25]. In this field, the frequently used PSs were confined to prototypical metal-to-ligand charge-transfer complexes [24,26–30], such as Ru(bpy)_3_^2+^ [31–34] and Ru(Phen)_3_^2+^ (Phen = 1,10-phenanthroline) [35–37], where their excited-state lifetime was usually <1 μs (τ = 600 ns for Ru(bpy)_3_^2+^ and 360 ns for Ru(Phen)_3_^2+^ in CH_3_CN) [36,38,39]. It will be a promising way to boost CO_2_ reduction via adjusting the excited-state population and lifetime of these PSs to improve their sensitizing ability. In the past decades, decreasing the energy level of organic ligands by implanting a conjugated group has been used as a common strategy for achieving a long-lived excited state, which can realize a transition from ^3^MLCT state to ^3^IL state (intraligand triplet excited state) [40–45]. In this field, we have first introduced ^3^IL-type PSs with a long-lived excited state into photocatalytic systems for efficient H_2_ production [43,45]. Unfortunately, the excited oxidation potentials of PSs usually become more positive in this process. This will greatly weaken the driving force for electron transfer from excited PSs to catalysts in thermodynamic catalytic processes [44,45]. As a result, how to substantially improve the sensitizing ability of molecular antenna whilst balancing its excited-state lifetime and redox driving force represents a key role in enhancing photoconversion efficiency, although it still remains a great challenge.

In this contribution, we put forward a new strategy to greatly boost photocatalytic CO_2_ reduction by improving the photosensitization ability of PSs. A family of Ru(II)-based PSs **Ru-2**, **Ru-3** and **Ru-4** were prepared by the selective addition of pyrene/pyrenyl ethynylene to the 3- and 5-positions of Phen in Ru(Phen)_3_^2+^ (**Ru-1**) (Supplementary Figs 1–18). The triplet lifetimes of these complexes were gradually prolonged and their excited-state oxidation potentials became less negative with fine-tuning the excited state from **Ru-1** with the ^3^MLCT state to **Ru-4** with the ^3^IL state, which provided a platform to compare the effect of PSs with different sensitizing abilities on photocatalytic CO_2_ reduction. Remarkably, the most efficient PS **Ru-3** simultaneously possesses a long triplet lifetime (68.2 μs), ∼189 times longer than that of Ru(Phen)_3_^2+^, and a suitable excited-state oxidation potential (−0.92 V *vs* Saturated Calomel Electrode (SCE)). Impressively, the sensitizing ability of **Ru-3** is >17 times higher than that of typical **Ru-1** and it can efficiently sensitize the dinuclear cobalt catalyst (**C-1**) for photochemical CO_2_-to-CO conversion with an extremely high TON of 66 480.

## RESULTS

### Molecular design and optical properties

**Figure 2.**
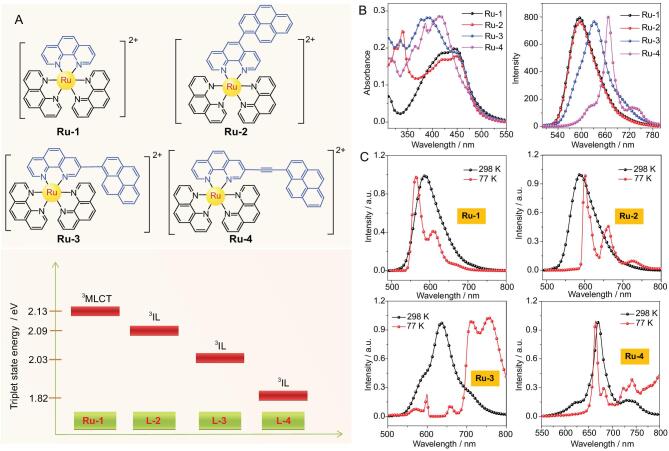
Fine-tuning the excited state of PSs to improve photosensitization for photochemical CO_2_-to-CO conversion. (A) Molecular structure of **Ru-1**–**Ru-4** (up). Energy diagram of triplet states of **Ru-1** and **L-2**–**L-4**, calculated at the B3LYP/6–31 G/genecp/LanL2DZ level with Gaussian 09 W (down). (B) UV-vis absorption and emission spectra of **Ru-1**–**Ru-4** under the same condition, *λ*_ex_ = 450 nm, c = 5.0 μM in deaerated CH_3_CN. (C) Emission spectra of Ru(II) complexes at RT and 77 K: **Ru-1**, *λ*_ex_ = 450 nm; **Ru-2**, *λ*_ex_ = 450 nm; **Ru-3**, *λ*_ex_ = 450 nm; **Ru-4**, *λ*_ex_ = 450 nm.

In order to achieve a more rational molecular design, Gaussian calculations were carefully performed to predict the energy level of the triplet states and molecular geometries of the pyrene–phen ligands **L-2**–**L-4** and **Ru-1**–**Ru-4** (Fig. 2, Supplementary Figs 19 and 20, and Supplementary Table 1). As shown in Fig. 2A, the triplet energy levels of these ligands were in the order of **L-2** > **L-3** > **L-4**, which was proportional to the dihedral angle between phen and pyrene (71° for **L-2** > 55° for **L-3** > 0° for **L-4**) (Supplementary Figs 19 and 20, and Supplementary Table 1). Meanwhile, the energy level of the ^3^IL states of these ligands was lower than that of the ^3^MLCT state of Ru(Phen)_3_^2+^, indicating that the triplet states of pyrene-functionalized Ru(II) complexes will be mainly localized on the pyrene–phen ligands (^3^IL states). Hence, fine-tuning the excited states is promising to be achieved by adjusting the position of pyrenyl on Phen of Ru(II) complexes.

According to Density Functional Theory (DFT) calculations, **Ru-2**–**Ru-4** were predicted as ideal models to determine the effect of the excited states on their sensitizing ability due to their well-proportioned energy-level gradient. All these complexes were synthesized according to the modified literature methods described in Supplementary Scheme 1 [42,46]. In this synthetic process, the precursors **L-2**, **L-3** and **L-4** were prepared by selective bromination of phen and

subsequent coupling with pyrenylboronic acid and ethynyl pyrene, which were used to coordinate with Ru^3+^ to generate corresponding **Ru-2**–**Ru-4** via a one-pot reaction, respectively. These complexes and intermediates were fully characterized by ^1^H NMR, ^13^C NMR and MS spectroscopy (Supplementary Figs 1–18).

The UV-vis absorption spectra of **Ru-1**–**Ru-4** are presented in Fig. 2B. **Ru-1** exhibits an absorption band of between 400 and 500 nm, corresponding to S_o_→^1^MLCT transition. For pyrene, a dual peak was observed at 319 and 334 nm, respectively, arising from π→π^*^ transition (Supplementary Fig. 21). The absorption spectrum of **Ru-2** is almost a superposition of that of **Ru-1** and pyrene, indicating a weak electron communication between Ru(Phen)_3_^2+^ and pyrenyl at the ground state [45]. In contrast to the absorption of **Ru-1** and **Ru-2**, a new peak at around 380 and 400 nm emerged in the absorption spectra of **Ru-3** and **Ru-4**, respectively, which resulted from the strong electronic interaction between the Ru center and pyrenyl. Interestingly, **Ru-3** and **Ru-4** exhibit a stronger visible-light-absorption ability than that of **Ru-1** and **Ru-2**. As a result, molecular regulation can gradually enhance the electron communication between Ru(Phen)_3_^2+^ and pyrenyl from **Ru-2** to **Ru-4**. Moreover, the absorption spectra of **Ru-1**–**Ru-4** in the presence of **C-1** or **TEOA** were almost similar to that of PSs alone (Supplementary Fig. 22). These results reveal that there is no intermolecular electronic interaction between the PSs under ground state and **C-1** (or **TEOA**) [45,47].

The photoluminescence (PL) spectra of **Ru-1**–**Ru-4** were carried out to investigate their excited-state properties (Fig. 2B and Supplementary Fig. 23). As shown in Supplementary Fig. 23, all the peaks in the PL spectra of **Ru-1**–**Ru-4** that emerged under the Ar atmosphere were significantly quenched as exposed to air, indicating the phosphorescence emission process, which mainly derived from the triplet state [48,49]. The PL spectrum of **Ru-1** showed a typical ^3^MLCT emission at 595 nm and a similar emission peak at 595 nm was also observed in the PL spectrum of **Ru-2**, manifesting that its ^3^MLCT characteristic in **Ru-2** partially remained after molecular regulation. In the PL spectra of **Ru-3** and **Ru-4**, a redshift and much broader phosphorescence emission at around 632 and 668 nm was presented in comparison with that of **Ru-1**. Especially for **Ru-4**, its emission reveals a fine structure and an obvious shoulder peak at 735 nm, indicating the existence of an ^3^IL emissive state. The emissive state of **Ru-3** should stand between those of **Ru-2** and **Ru-4**. All the above results were further illuminated by 77 K emission and nanosecond transient absorption spectra.

The emission spectra of **Ru-1**–**Ru-4** at 77 and 298 K were compared for clarifying their emissive state (Fig. 2C). A large hypochromic shift between 77 and 298 K spectra was observed for **Ru-1** (Δ*E*_s_, 665.7 cm^−1^), indicating a typical ^3^MLCT emissive state [48,50]. By contrast, **Ru-4** showed a small hypochromic shift at 670 nm (148.7 cm^−1^), suggesting an ^3^IL emissive state. For **Ru-2**, an emission band between 600 and 800 nm was observed at 77 K, which matched well with the phosphorescence of pyrene [51]. Thus, this indicated a pyrenyl localized emissive state. Interestingly, the PL spectrum of **Ru-3** exhibits multiple emission peaks at 77 K. Two weak peaks at around 600 and 660 nm close to the pyrenyl localized emissive state of **Ru-2** were detected in the PL spectrum of **Ru-3** and a strong dual emission around 750 nm that corresponded to the ^3^IL emissive state was also observed. This dual emission also emerged in the PL spectrum of **Ru-4**. As a result, it can be proposed that the emissive state of **Ru-3** was between those of **Ru-2** and **Ru-4**, but was dominated by the ^3^IL emissive state.

**Figure 3.**
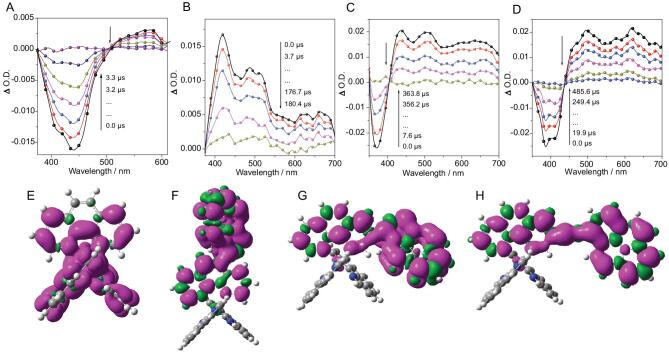
The triplet excited-state population of PSs. Nanosecond time-resolved transient difference absorption spectra of (A) **Ru-1**, (B) **Ru-2**, (C) **Ru-3** and (D) **Ru-4**. After pulsed excitation at 450 nm for **Ru-1** and **Ru-4**, 355 nm for **Ru-2** and **Ru-3** (c_PS_ = 5 μM in deaerated CH_3_CN). Spin-density surface of (E) **Ru-1**, (F) **Ru-2**, (G) **Ru-3** and (H) **Ru-4**. Calculated at the B3LYP/6–31 G/genecp/LanL2DZ level with Gaussian 09 W.

To further decipher their excited-state properties, nanosecond transient absorption spectra were performed on these four PSs, **Ru-1**–**Ru-4** (Fig. 3A–D). The transient spectrum of **Ru-1** showed a bleaching band at around 450 nm corresponding to the depletion of the ground state, which was a typical characteristic of the ^3^MLCT state. In the transient spectra of **Ru-2**–**Ru-4**, there was some superposition between the bleaching band and the excited-state absorption band [48]. **Ru-2** only afforded a transient absorption band between 380 and 700 nm, along with its ^3^MLCT emission at 595 nm; thus, its excited state could be ascribed to a mix of ^3^MLCT and the pyrenyl localized excited state. The transient spectrum of **Ru-3** shows a positive absorption above 400 nm and a bleaching peak at 371 nm, manifesting a feature of the ^3^IL state. For **Ru-4**, a strong bleaching band at around 400 nm was observed, which matched well with its steady absorption to re-affirm its ^3^IL state.

Spin-density surfaces of these Ru(II) complexes (**Ru-1**–**Ru-4**) were calculated at B3LYP/6–31 G/genecp/LanL2DZ level with Gaussian 09, which can rationalize the population of their triplet states. As shown in Fig. 3, the spin density of **Ru-1** was primarily localized on the Ru-Phen coordination center, indicative of a ^3^MLCT state. For **Ru-2**–**Ru-4**, their spin densities were mainly distributed on the Phen and pyrene owing to the lower triplet energy level of pyrenyl-Phen than that of the ^3^MLCT state. These results supported that the excited states of **Ru-2**–**Ru-4** were largely populated on the ligands, which matched well with the experimental results. Therefore, we proposed that the redox center should be localized at pyrenyl functionalized ligands. All the photophysical data are summarized in Table 1 and the combination of these data with these calculated results can illustrate that fine-tuning of the excited state of these PSs from ^3^MLCT state to the ^3^IL state was realized. This provided a platform on which to compare the effect of PSs with different sensitizing abilities on photocatalytic CO_2_ reduction.

**Table 1.** Summary of photophysical data of **Ru-1**–**Ru-4**.^a^

	λ_abs_/nm	λ_em_/nm	ϵ/(M^−1^ cm^−1^)	τ/μs^b^	K_1_/(M^−1^)^c^
**Ru-1**	447	595	20 914	0.4	375
**Ru-2**	447	595	19 824	32.0	957
**Ru-3**	391	632	33 318	68.2	3239
**Ru-4**	415	668	49 808	118.7	4419

^a^5.0 μM **Ru-1**–**Ru-4** in CH_3_CN.

^b^Triplet excited-state lifetime measured by transient absorption.

^c^Stern–Volmer-quenching constants with **C-1** as the quenchers.

### Photocatalytic CO_2_ reduction

**Figure 4.**
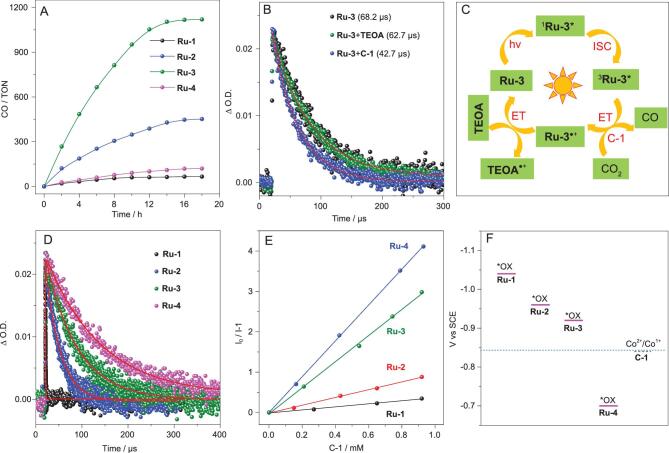
Photocatalytic CO_2_ reduction and photocatalytic mechanism. (A) Photocatalytic CO_2_ reduction with **Ru-1**–**Ru-4** under irradiation of a LED light (450 nm, 100 mW cm^−2^, irradiation area, 0.8 cm^2^) in the presence of PS (20 μM), **C-1** (1.0 μM) and **TEOA** (0.3 M) in 5 mL CO_2_-saturated CH_3_CN/H_2_O (*v*/*v* = 9/1) solution. Each photocatalytic reaction was repeated at least three times. (B) Nanosecond transient absorption spectra of kinetic decay trace of **Ru-3** (black), **Ru-3** with 0.4 mM **TEOA** (green), and **Ru-3** with 0.5 μM **C-1** (blue). (C) Photocatalytic process with **Ru-3** as PS. ET, electron transfer; ISC, intersystem crossing. (D) Kinetic traces of **Ru-1**–**Ru-4** followed at 450 nm. (E) Stern−Volmer plot of **Ru-1**−**Ru-4**. λ_ex_ = 450 nm, c_PS_ = 5.0 μM. (F) Energy diagram depicting the excited-state oxidation potential (*Ox) of **Ru-1**–**Ru-4** and the onset reduction potential of **C-1** in the catalytic system.

Further, to reveal the influence of different sensitizing abilities on CO_2_ reduction, photocatalytic activities of these Ru(II) complexes (**Ru-1**–**Ru-4**) were investigated in 5 mL CO_2_-saturated CH_3_CN/H_2_O (v/v = 9/1) solution containing **C-1** as the catalyst and **TEOA** as the electron sacrificial agent under the illumination of a 450 nm LED (Fig. 4 and Supplementary Tables 2–4). As shown in Fig. 4A, the TON of **C-1** with **Ru-3** was up to 1120, 17 times higher than that with **Ru-1**. In the **Ru-3**-containing photocatalyic system, the TON towards **C-1** can reach as high as 66 480 under the optimized condition, which substantially exceeds our previously reported results with **Ru-1** as the PS (TON = 16 896, Supplementary Table 3) [35]. In addition, no or a trace amount of CO was detected in the absence of PS, **TEOA**, **C-1**, light or CO_2_, manifesting that all the above factors are indispensable for efficient photocatalytic CO_2_ reduction (Supplementary Table 4).

The photocatalytic mechanism was studied by steady and transient quenching experiments (Fig. 4). Phosphorescence quenching experiments of **Ru-1**–**Ru-4** reveal that their excited states can be efficiently quenched by **C-1**; nevertheless, no change on their photoluminescence spectra was observed in the presence of **TEOA** (Supplementary Figs 24 and 25). We therefore proposed that the photocatalytic process was dominated by the oxidation mechanism

for **Ru-1**–**Ru-4**-containing systems [35,36], which was further confirmed by nanosecond transient absorption. The triplet lifetimes of **Ru-1**–**Ru-4** remained before and after adding to **TEOA**, but became shorter in the presence of **C-1**, confirming that the initial step for electron transfer should be from excited PSs to **C-1** (Fig. 4B and Supplementary Fig. 26). As a result, all these photocatalytic systems could be determined as oxidation mechanisms (Fig. 4C).

With the above results in mind, both triplet excited-state lifetimes and the excited-state oxidation potentials of PSs are key factors affecting electron-transfer efficiency in photocatalytic systems. From the view of kinetics, the long-lived triplet state of PSs greatly contributed to intermolecular electron transfer/energy transfer. Thus, Stern–Volmer-quenching constants of PSs by **C-1** were in the order of 4.4 × 10^3^ M^−1^ for **Ru-4** > 3.2 × 10^3^ M^−1^ for **Ru-3** > 9.6 × 10^2^ M^−1^ for **Ru-2** > 3.8 × 10^2^ M^−1^ for **Ru-1**, which was proportional to their excited-state lifetimes (Fig. 4D and E). From the thermodynamics viewpoint, the excited-state oxidation potentials of PSs determine the driven force of electron transfer from excited PSs to **C-1** (Supplementary Table 5). As shown in Fig. 4F, the absolute value of the excited-state oxidation potential was in the order of **Ru-4** < **C-1** < **Ru-3** < **Ru-2** < **Ru-1**, indicating that excited **Ru-1**–**Ru-3** can provide a sufficient driven force for delivering the electron to **C-1** but the driven force from excited **Ru-4** to **C-1** was humble. Notably, the triple bond in **Ru-4** makes greater delocalization than **Ru-2** and **Ru-3**, indicating a lower excited-state energy level of the ligand in **Ru-4**. This delocalization can contribute to obtaining a long-lived excited state. However, the oxidizing ability of excited **Ru-4** becomes weaker, which is disadvantageous for electron transfer from excited **Ru-4** to **C-1** and will further decrease the photocatalytic

activity. As a result, **Ru-3**, as a trade-off PS, possesses a long-lived triplet state and a suitable excited-state oxidation potential simultaneously, highlighting its great potential for efficient CO_2_ reduction.

## CONCLUSION

In summary, we have developed a novel strategy to boost photocatalytic CO_2_ reduction via improving photosensitization. In this work, four Ru-based complexes (**Ru-1**–**Ru-4**) were prepared and they presented a gradual variable excited state from **Ru-1** with the ^3^MLCT state to **Ru-4** with the ^3^IL state, which provides a platform on which to compare the effect of PSs with different sensitizing abilities on photocatalytic CO_2_ reduction. Remarkably, the catalytic activity of **Ru-3** was >17 times higher than that of **Ru-1** and the TON towards **C-1** can reach 66 480. The outstanding photocatalytic activity of **Ru-3** was chiefly ascribed to its long-lived excited state and suitable excited-state oxidation potential. This work provides a new insight into substantially improving visible-light-driven CO_2_-reduction performance through fine-tuning the excited states of PSs on a molecular level.

## METHODS

### Materials and instrumentation

All the reactions were performed in argon unless otherwise mentioned. All the solvents were of analytical grade and distilled before use. The dichloro(p-cymene)ruthenium(II) dimer and 1,10-Phenanthroline were purchased from Sigma-Aldrich. The 1-pyrenylboronic acid, NH_4_PF_6_ and K_2_CO_3_ were purchased from HEOWNS. The Tetrakis(triphenylphosphine)palladium(0) and CuI were purchased from Adamas-beta. Chromatographic-grade acetonitrile was purchased from Adamas Reagent. The synthetic scheme of **Ru-1**–**Ru-4** is presented in Supplementary Scheme 1. The synthetic intermediates and target complexes were evidenced by ^1^H NMR, ^13^C NMR and mass spectroscopy. Elemental analysis was performed as C/H/N analyses on the Vario EL Cube (Elementar, Germany).

Electrochemical measurements were carried out on a CHI 760E electrochemical workstation at room temperature. The amount of CO product was analysed by gas chromatography (Shimadzu GC-2014+AT 230C, TDX-01 column, TCD, argon carrier). UV-vis absorption spectra were recorded on a LAMBDA750 UV-vis spectrophotometer. Fluorescence spectra were taken on a Hitachi F4600 spectrofluorometer. Transient absorption spectra were measured on the LP980 laser flash photolysis instrument (Edinburgh, UK).

### Photocatalytic CO_2_ reduction

Photocatalytic CO_2_ reduction was conducted under 1 atm of CO_2_ at 25°C in 5 mL reactor containing PS (20.0 μM), catalyst (1.0 μM), **TEOA** (0.3 M), 0.5 mL H_2_O and 4.5 mL CH_3_CN. The photocatalytic system was bubbled with CO_2_ for 30 min. The mixture was continuously stirred and irradiated under a LED (*λ* = 450 nm, 100 mW cm^−2^).

### DFT calculation

The geometries and the spin-density surfaces of the complexes (**Ru-1**–**Ru-4**) were performed at the B3LYP/6–31 G/LanL2DZ level. There are no imaginary frequencies for all optimized structures of **L-1**–**L-4** and **Ru-1**–**Ru-4**. The triplet-state energy levels were carried out with the time-dependent DFT (TDDFT) method. All these calculations were performed with Gaussian 09 W.

## DATA AND SOFTWARE AVAILABILITY

All data needed to evaluate the conclusions in the paper are present in the paper and/or the Supplementary Materials. Additional data related to this paper may be requested from the authors.

## Supplementary Material

nwaa112_Supplemental_Revised
