# Supplementary material for: Improving photosensitization for photochemical CO2-to-CO conversion
Source: Natl Sci Rev. 2020 May 28;7(9):1459–67. doi: 10.1093/nsr/nwaa112 (PMC8288749; doi:10.1093/nsr/nwaa112)
Supplement: nwaa112_Supplemental_Revised [file nwaa112_supplemental_revised.doc]

Supplementary Information

**Improving Photosensitization for Photochemical CO_2_-to-CO Conversion**

*Ping Wang,^1^ Ru Dong, ^1^ Song* *Guo^1^*, Jianzhang Zhao, ^2^ Zhi-Ming Zhang, ^1^* and Tong-Bu Lu^1^*

^1^MOE International Joint Laboratory of Materials Microstructure, Institute for New Energy Materials and Low Carbon Technologies, School of Materials Science and Engineering, Tianjin University of Technology, Tianjin 300384, China.

^2^State Key Laboratory of Fine Chemicals, School of Chemical Engineering, Dalian University of Technology, Dalian 116024 China

*E-mail: guosong@email.tjut.edu.cn; zmzhang@email.tjut.edu.cn

**Table of Contents**

[**Experimental Section** 1](#__RefHeading___Toc43905937)

[**Materials and methods.** 1](#__RefHeading___Toc43905938)

[**Instruments**. 1](#__RefHeading___Toc43905939)

[**Photocatalytic CO_2_ reduction.** 1](#__RefHeading___Toc43905940)

[**DFT calculation.** 1](#__RefHeading___Toc43905941)

[**Synthetic process of Ru-2** – **Ru-4** 2](#__RefHeading___Toc43905942)

[**Supplementary Scheme 1.** Synthetic route of **Ru-1** – **Ru-4**. 2](#__RefHeading___Toc43905943)

[**Synthesis of 1.** 2](#__RefHeading___Toc43905944)

[**Synthesis of L-2.** 2](#__RefHeading___Toc43905945)

[**Synthesis of Ru-2.** 3](#__RefHeading___Toc43905946)

[**Synthesis of 2.** 3](#__RefHeading___Toc43905947)

[**Synthesis of L-3.** 4](#__RefHeading___Toc43905948)

[**Synthesis of Ru-3.** 4](#__RefHeading___Toc43905949)

[**Synthesis of 4.** 4](#__RefHeading___Toc43905950)

[**Synthesis of L-4.** 5](#__RefHeading___Toc43905951)

[**Synthesis of Ru-4.** 5](#__RefHeading___Toc43905952)

[**Supplementary Scheme 2.** Molecular structure of **Ru-1** – **Ru-4**, **C-1** and **TEOA**. 6](#__RefHeading___Toc43905953)

[**Structural characterization** 7](#__RefHeading___Toc43905954)

[**Supplementary Figure 1.** ^1^H NMR spectrum of **1** (400 MHz, CDCl_3_). 7](#__RefHeading___Toc43905955)

[**Supplementary Figure 2.** ^1^H NMR spectrum of **2** (400 MHz, CDCl_3_). 7](#__RefHeading___Toc43905956)

[**Supplementary Figure 3.** ^1^H NMR spectrum of **4** (400 MHz, CDCl_3_). 8](#__RefHeading___Toc43905957)

[**Supplementary Figure 4.** ^1^H NMR spectrum of **L-2** (400 MHz, CDCl_3_). 8](#__RefHeading___Toc43905958)

[**Supplementary Figure 5.** TOF HRMS ESI of **L-2**. 9](#__RefHeading___Toc43905959)

[**Supplementary Figure 6.** ^1^H NMR spectrum of **L-3** (400 MHz, CDCl_3_). 9](#__RefHeading___Toc43905960)

[**Supplementary Figure 7.** TOF HRMS ESI of **L-3**. 10](#__RefHeading___Toc43905961)

[**Supplementary Figure 8.** ^1^H NMR spectrum of **L-4** (400 MHz, CDCl_3_). 10](#__RefHeading___Toc43905962)

[**Supplementary Figure 9.** TOF HRMS ESI of **L-4**. 11](#__RefHeading___Toc43905963)

[**Supplementary Figure 10.** ^1^H NMR spectrum of **Ru-2** (400 MHz, *d^6^*-acetone). 11](#__RefHeading___Toc43905964)

[**Supplementary Figure 11.** ^13^C NMR spectrum of **Ru-2** (100 MHz, *d^6^*-acetone). 12](#__RefHeading___Toc43905965)

[**Supplementary Figure 12.** TOF HRMS ESI of **Ru-2**. 12](#__RefHeading___Toc43905966)

[**Supplementary Figure 13.** ^1^H NMR spectrum of **Ru-3** (400 MHz, DMSO-*d^6^*). 13](#__RefHeading___Toc43905967)

[**Supplementary Figure 14.** ^13^C NMR spectrum of **Ru-3** (100 MHz, *d^6^*-acetone). 13](#__RefHeading___Toc43905968)

[**Supplementary Figure 15.** TOF HRMS ESI of **Ru-3**. 14](#__RefHeading___Toc43905969)

[**Supplementary Figure 16.** ^1^H NMR spectrum of **Ru-4** (400 MHz, DMSO-*d^6^*). 14](#__RefHeading___Toc43905970)

[**Supplementary Figure 17.** ^13^C NMR spectrum of **Ru-4** (100 MHz, *d^6^*-acetone). 15](#__RefHeading___Toc43905971)

[**Supplementary Figure 18.** TOF HRMS ESI of **Ru-4**. 15](#__RefHeading___Toc43905972)

[**DFT calculations.** 16](#__RefHeading___Toc43905973)

[**Supplementary Figure 19.** 16](#__RefHeading___Toc43905974)

[**Supplementary Table 1.** 17](#__RefHeading___Toc43905975)

[**Supplementary Figure 20.** 17](#__RefHeading___Toc43905976)

[**UV-vis absorption and Phosphorescence emission spectra.** 18](#__RefHeading___Toc43905977)

[**Supplementary Figure 21.** 18](#__RefHeading___Toc43905978)

[**Supplementary Figure 22.**. 18](#__RefHeading___Toc43905979)

[**Supplementary Figure 23.** 19](#__RefHeading___Toc43905980)

[**Photocatalytic CO_2_ reduction** 20](#__RefHeading___Toc43905981)

[**Supplementary Table 2.** 20](#__RefHeading___Toc43905982)

[**Supplementary Table 3.** 20](#__RefHeading___Toc43905983)

[**Supplementary Table 4.** 20](#__RefHeading___Toc43905984)

[**Supplementary Figure 24.**. 21](#__RefHeading___Toc43905985)

[**Supplementary Figure 25.**. 21](#__RefHeading___Toc43905986)

[**Supplementary Figure 26.** 22](#__RefHeading___Toc43905987)

[**Cyclic voltammograms** 23](#__RefHeading___Toc43905988)

[**Supplementary Table 5.** 23](#__RefHeading___Toc43905989)

**Experimental Section**

**Materials and methods.**

All the reactions were performed in argon unless otherwise mentioned. All the solvents were analytical grade and distilled before the use. The dichloro(p-cymene)ruthenium(II) dimer and 1,10-Phenanthroline was purchased from Sigma-Aldrich. The 1-pyrenylboronic acid, NH_4_PF_6_, and K_2_CO_3_ were purchased from HEOWNS. The Tetrakis(triphenylphosphine)palladium(0) and CuI were purchased from Adamas-beta. Chromatographic grade acetonitrile was purchased from Adamas Reagent. The synthetic scheme of **Ru-1** – **Ru-4** is presented in Scheme S1. The synthetic intermediates and target complexes were evidenced by ^1^H NMR, ^13^C NMR and mass spectroscopy.

Instruments.

Electrochemical measurements were carried out on a CHI 760E electrochemical workstation at room temperature. The amount of CO product was analyzed by gas chromatography (Shimadzu GC-2014+AT 230C, TDX-01 column, TCD, argon carrier). UV-vis absorption spectra were recorded on a LAMBDA750 UV-vis spectrophotometer. Fluorescence spectra were taken on Hitachi F4600 spectrofluorometer. Transient absorption spectra were measured on the LP980 laser flash photolysis instrument (Edinburgh, U.K.).

**Photocatalytic CO_2_ reduction.**

Photocatalytic CO_2_ reduction was conducted under 1 atm of CO_2_ at 25 °C in 5 mL reactor containing PS (2 × 10^-5^ M), catalyst (1 × 10^-6^ M), **TEOA** (0.3 M), 0.5 mL H_2_O and 4.5 mL CH_3_CN. The photocatalytic system was bubbled with CO_2_ for 30 min. The mixture was continuously stirred and irradiated under a LED (*λ* = 450 nm, 100 mW·cm^-2^).

**DFT calculation.**

The geometries and the spin density surfaces of the complexes (**Ru-1** – **Ru-4**) were performed at the B3LYP/6-31G/LanL2DZ level. There are no imaginary frequencies for all optimized structures of **L-1** **–** **L-4** and **Ru-1 – Ru-4**. The triplet state energy levels were carried out with the time-dependent DFT (TDDFT) method. All these calculations were performed with Gaussian 09W.

**Synthetic process of Ru-2** – **Ru-4**


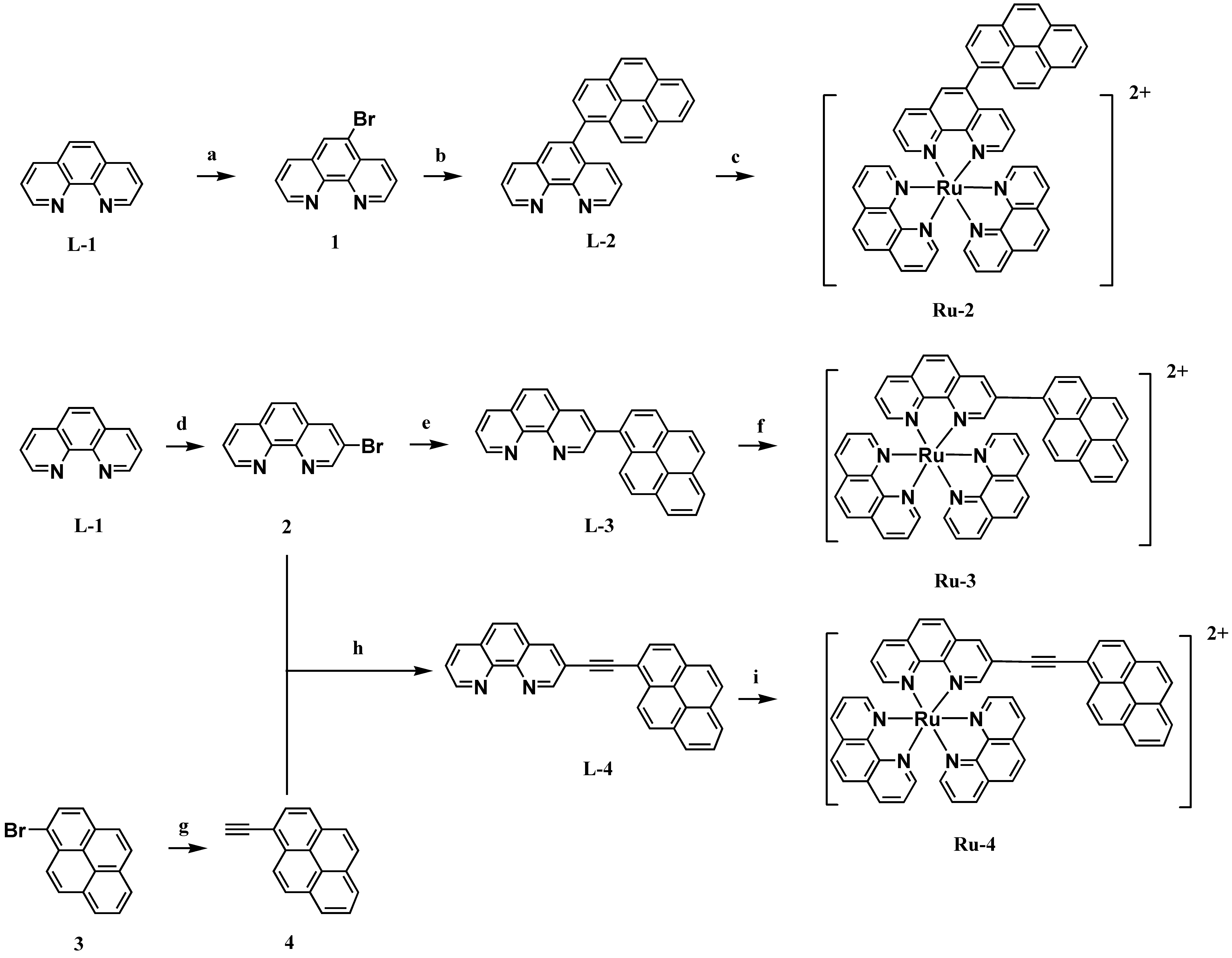


**Supplementary Scheme 1.** Synthetic route of **Ru-1** – **Ru-4**.

**Synthesis of 1.**

1,10-Phenanthroline (**L-1**) (0.36 g, 2 mmol) was placed in a heavy-walled glass reaction tube with a Teflon screw top fitted. 1.2 mL of oleum (15 %) and bromine (0.06 mL, 1.16 mmol) were added to reaction vessel. Then, the temperature was slowly raised to 135 °C. After 23 h reaction, the mixture was cooled down to room temperature, and then poured into ice water. The resulting mixture was neutralized with NH_4_OH. The mixture was extracted with CHCl_3_. The extracts were stirred with charcoal and then dried over Na_2_SO_4_. The crude mixture was recrystallized from hot diethyl ether with a minimum amount of CH_2_Cl_2_ to yield target product. (Yield: 0.47 g, 90.1 %). ^1^H NMR (400 MHz, CDCl_3_) δ 9.23 – 9.22 (m, 2H), 8.68 (dd, *J* = 8.3, 1.6 Hz*,* 1H), 8.22 – 8.17 (m, 2H), 7.78 – 7.75 (m, 1H), 7.69 – 7.66 (m, 1H).

**Synthesis of L-2.**

5-Bromo-1,10-phenanthroline (**1**) (191.7 mg, 0.74 mmol) and 1-pyrene boronic acid (199.6 mg, 0.81 mmol) were dissolved in a mixed solution (30 mL toluene and 10 mL ethanol). The yellow solution was degassed with Ar for 30 min. Then a saturated aqueous solution of Ba(OH)_2_ (40 mL) was added and the biphasic mixture was degassed for an additional 30 min. After adding Pd(PPh_3_)_4_ (34.7 mg, 0.03 mmol), the mixture was refluxed with vigorous stirring under Ar for 2 days. As cooling down to room temperature, the aqueous layer was extracted with toluene (3 × 10 mL) and the combined organic solution were washed with water for three times (3 × 20 mL). The result solution was dried over MgSO_4_, filtered, and rotary evaporated to yield brown oily residue. After crystallization, the light yellow powder was collected by vacuum filtration on a Buchner funnel. Yield: 194.0 mg, 62.9 %. ^1^H NMR (400 MHz, CDCl_3_) δ 9.31 (dd, *J* = 4.4, 1.6 Hz, 1H), 9.23 (dd, *J* = 4.2, 1.6 Hz, 1H), 8.34 (d, *J* = 7.8 Hz, 2H), 8.20 – 8.17 (m, 3H), 8.08 – 8.04 (m, 2H), 7.98 – 7.92 (m, 2H), 7.79 – 7.73 (m, 2H), 7.63 (d, *J* = 9.3 Hz, 1H), 7.49 – 7.42 (m, 2H). HRMS (ESI): (C_28_H_16_N_2_): calcd *m / z* = 380.1313, found *m / z* = 381.1413.

**Synthesis of Ru-2.**

[RuCl_2_(cymene)]_2_ (40.2 mg, 0.065 mmol) and 5-pyrenyl-1,10-phenanthroline (**L-2**) (49.5 mg, 0.13 mmol) were dissolved in ethanol (5 mL) and the mixture was stirred for 2 h at Ar atmosphere. Then 10 mL water and 1,10-phenanthroline (**L-1**) (46.9 mg, 0.26 mmol) were added to the above solution, which was then refluxed for additional 24 h. After cooling down to room temperature, the solution was concentrated under reduced pressure and treated with a saturated aqueous solution of NH_4_PF_6_ to give a red precipitate. The crude product was purified by column chromatography (silica gel, eluted with acetonitrile : water : saturated aqueous NaNO_3_ = 100 : 9 : 1, v/v/v). After addition of a saturated aqueous solution of NH_4_PF_6_, the red precipitate formed, which was washed with water and dried in air to yield red solid. Yield: 45.9 mg, 31.1 %. ^1^H NMR (400 MHz, acetone-*d^6^*) δ 8.89 – 8.79 (m, 5H), 8.62 (dd, *J* = 5.2, 0.9 Hz, 1H), 8.58 (d, *J* = 1.4 Hz, 1H), 8.55 – 8.48 (m, 3H), 8.47 – 8.41 (m, 7H), 8.36 – 8.34 (m, 2H), 8.28 – 8.23 (m, 1H), 8.18 – 8.00 (m, 4H), 7.96 – 7.74 (m, 6H), 7.68 – 7.62 (m, 2H). ^13^C NMR (100 MHz, acetone-*d^6^*) δ 154.41, 154.34, 154.11, 154.06, 153.98, 149.42, 149.36, 149.03, 148.99, 148.97, 140.55, 137.95, 137.91, 136.81, 136.77, 132.80, 132.50, 132.37, 132.05, 131.76, 131.67, 130.76, 130.67, 130.58, 129.65, 129.44, 129.37, 129.28, 129.15, 128.33, 127.62, 127.59, 127.46, 127.21, 127.16, 127.07, 126.94, 126.60, 125.88, 125.84, 125.72, 125.51, 125.43, 125.23. HRMS (ESI): (C_52_H_32_N_6_Ru) ^2+^: calcd *m / z* = 421.0866, found *m / z* = 421.0875. Anal. Calcd for C_52_H_32_F_12_N_6_P_2_Ru: C, 55.18; H, 2.85; N, 7.43. Found: C, 55.21; H, 2.92; N, 7.34.

**Synthesis of 2.**

A solution of 1,10-Phenanthroline (**L-1**) (1.0 g, 4.30 mmol) in nitrobenzene (2 mL) was heated to 140 ^o^C in a 250 mL 3-neck flask. Bromine (6.4 mmol in 0.93 ml nitrobenzene) was added dropwise over a period of 1 h. After stirring for 3 h at the same temperature, the reaction mixture was cooled down to room temperature, treated with concentrated ammonium hydroxide (10 mL) and extracted with dichloromethane (3 × 50 mL). The combined organic layers were washed with water (3 × 50 mL) and dried with MgSO_4_. Concentration in vacuum afforded a suspension of the products in nitrobenzene. The nitrobenzene was removed by dissolving the suspension in dichloromethane (10 mL) and filtering it through silica gel (30 mL) using dichloromethane as the eluent. After the nitrobenzene eluted out, the products were recovered by gradually increasing the polarity of the eluent up to 10 % MeOH in CH_2_Cl_2_ to obtain white powders of **2**. Yield: 0.36 g, 33.2 %. ^1^ H NMR (400 MHz, CDCl_3_) δ 9.23 − 9.20 (m, 2H), 8.43 (d, *J* =2.0 Hz, 1H), 8.30 (d, *J* =8.0 Hz, 1H), 7.86 (d, *J* =8.8 Hz, 1H), 7.76 − 7.68 (m, 2H).

**Synthesis of L-3.**

Compound **L-3** was synthesized by a similar method as 5-pyrenyl-1,10-phenanthroline (**L-2**) except that 5-Bromo-1,10-phenanthroline (**1**) (191.7 mg, 0.74 mmol) was replaced as 3-Bromo-1, 10-phenanthroline (**2**) (191.7 mg, 0.74 mmol). The oil was triturated with petroleum ether to give an olive drab precipitate. Yield: 230.3 mg, 81.8 %. ^1^H NMR (400 MHz, CDCl_3_) δ 9.52 (d, *J* = 1.6 Hz, 1H), 9.31 (d, *J* = 3.6 Hz, 1H), 8.54 (d, *J* = 1.8 Hz, 1H), 8.38 – 8.31 (m, 2H), 8.27 – 8.04 (m, 8H), 7.94 (q, *J* = 8.8 Hz, 2H), 7.75 – 7.72 (m, 1H). HRMS (ESI): (C_28_H_16_N_2_): calcd *m / z* = 380.1313, found *m / z* = 381.1447.

**Synthesis of Ru-3.**

The complex was synthesized by a similar method as **Ru-2** except that 5-pyrenyl-1,10-phenanthroline (**L-2**) (49.5 mg, 0.13 mmol) was replaced as 3-pyrenyl-1,10-phenanthroline (**L-3**) (49.5 mg, 0.13 mmol). Red solid was obtained With the yield of 61.8 mg (42.0 %). ^1^H NMR (400 MHz, DMSO-*d^6^*) δ 9.14 (s, 1H), 8.91 – 8.79 (m, 4H), 8.55 – 8.35 (m, 11H), 8.28 – 8.07 (m, 9H), 7.88 – 7.73 (m, 5H), 7.54 (t, *J* = 12.4 Hz, 1H), 7.16 (s, 1H). ^13^C NMR (400 MHz, acetone-*d^6^*) δ 154.94, 154.50, 154.29, 154.26, 154.20, 154.17, 149.06, 149.01, 148.98, 147.87, 139.76, 138.49, 138.01, 137.96, 137.90, 137.68, 132.71, 132.23, 132.09, 132.04, 132.02, 131.91, 131.89, 131.46, 129.56, 129.53, 129.42, 129.31, 129.12, 129.06, 128.11, 127.61, 127.32, 127.14, 127.11, 127.05, 126.90, 126.53, 125.99, 125.41, 125.07, 123.62. HRMS (ESI): (C_52_H_32_N_6_Ru)^2+^: calcd *m / z* = 421.0866, found *m / z* = 421.0885. Anal. Calcd for C_52_H_32_F_12_N_6_P_2_Ru: C, 55.18; H, 2.85; N, 7.43. Found: C, 55.16; H, 2.96; N, 7.33.

**Synthesis of 4.**

1-bromopyrene (**3**) (539.7 mg, 1.92 mmol) and trimethylsilylacetylene (410.0 mg, 4.18 mmol) were dissolved in a mixed solvent (25.0 mL tetrahydrofuran and 20.0 mL triethylamine). The above solution was degassed for 30 min, and then CuI (0.01 g, 0.05 mmol) and Bis(triphenylphosphine) palladium chloride (270.2 mg, 0.30 mmol) were added. The mixture was refluxed for 24 h under dark condition. The resulting solution was poured into water and extracted with methylene chloride and ethyl acetate, dried over anhydrous MgSO_4_. The solvent was removed with a rotary evaporator, and the crude intermediate product was purified by column chromatography using petroleum ether as eluent to yield the intermediate product (260.0 mg, 45.4 %). Then the intermediate product (260.0 mg, 0.87 mmol) and potassium fluoride (500.4 mg, 8.6 mmol) were dissolved in 60 mL ethyl alcohol. After refluxed for 12 h, the resulting solution was poured into water and extracted with methylene chloride and ethyl acetate and dried over anhydrous MgSO_4_. The solvent was removed with a rotary evaporator, and the crude product was purified by silica gel column chromatography using petroleum ether as eluent to yield **4** (0.16 g, 81.2 %). Yield: 160.0 mg, 81.2 %. ^1^H NMR (400 MHz, CDCl_3_) δ 8.59 (d, *J* = 9.1 Hz, 1H), 8.24 – 8.16 (m, 4H), 8.11 (d, *J* = 8.4 Hz, 2H), 8.06 – 8.02 (m, 2H), 3.63 (s, 1H).

**Synthesis of L-4.**

A mixture of 3-bromo-1,10-phenanthroline (**2**) (101.0 mg, 0.39 mmol), 1-ethynylpyrene (131.3 mg, 0.58 mmol), [Pd(PPh_3_)_4_] (67.0 mg, 0.058 mmol), and n-propylamine (15 mL) was stirred at 80 ^o^C under Ar atmosphere for 2 days. After cooling down to room temperature, the precipitate was filtered, and purified by silica gel column chromatography using CH_2_Cl_2_ / MeOH (99/1, v/v) as eluent to yield **L-4** (125.1 mg, 79.2 %) as a brownish solid. Yield: 125.0 mg, 79.2 %. ^1^H NMR (400 MHz, CDCl_3_) δ 9.45 (s, 1H), 9.23 (s, 1H), 8.71 (d, *J* = 8.9 Hz, 1H), 8.53 (s, 1H), 8.29 – 8.03 (m, 9H), 7.82 (s, 2H), 7.67 – 7.65 (m, 1H). HRMS (ESI): (C_30_H_16_N_2_): calcd *m / z* = 404.1313, found *m / z* = 405.1394.

**Synthesis of Ru-4.**

The complex was synthesized by a similar method as **Ru-2** except that 5-pyrenyl-1,10-phenanthroline (**L-2**) (49.5 mg, 0.13 mmol) was replaced as 3-(Pyren-1’-yl)ethynyl-1,10-phenanthroline (**L-4**) (52.6 mg, 0.13 mmol). Red solid was obtained. Yield: 58.8 mg, 39.1 %. ^1^H NMR (400 MHz, DMSO-*d^6^*) δ 9.25 (d, *J* = 1.4 Hz, 1H), 8.89 (d, *J* = 7.5 Hz, 2H), 8.83 – 8.78 (m, 4H), 8.50 – 8.31 (m, 14H), 8.27 – 8.18 (m, 4H), 8.12 (t, *J* = 9.9 Hz, 1H), 8.02 (d, *J* = 5.0 Hz, 1H), 7.90 – 7.76 (m, 5H). ^13^C NMR (400 MHz, acetone-*d^6^*) δ 155.67, 154.55, 154.45, 154.35, 154.22, 154.02, 149.16, 149.14, 148.95, 148.93, 148.86, 147.83, 139.50, 137.98, 133.24, 132.92, 132.41, 132.13, 132.08, 132.02, 131.78, 131.40, 130.60, 130.10, 129.97, 129.19, 129.16, 129.13, 128.86, 128.11, 127.80, 127.37, 127.33, 127.30, 127.16, 127.11, 125.77, 125.42, 125.01, 124.70, 123.19, 116.26, 95.32, 91.25. HRMS (ESI): (C_54_H_32_N_6_Ru)^2+^: calcd *m / z* = 443.0866, found *m / z* = 443.0876. Anal. Calcd for C_54_H_32_F_12_N_6_P_2_Ru: C, 56.11; H, 2.79; N, 7.27. Found: C, 56.01; H, 2.88; N, 7.19.


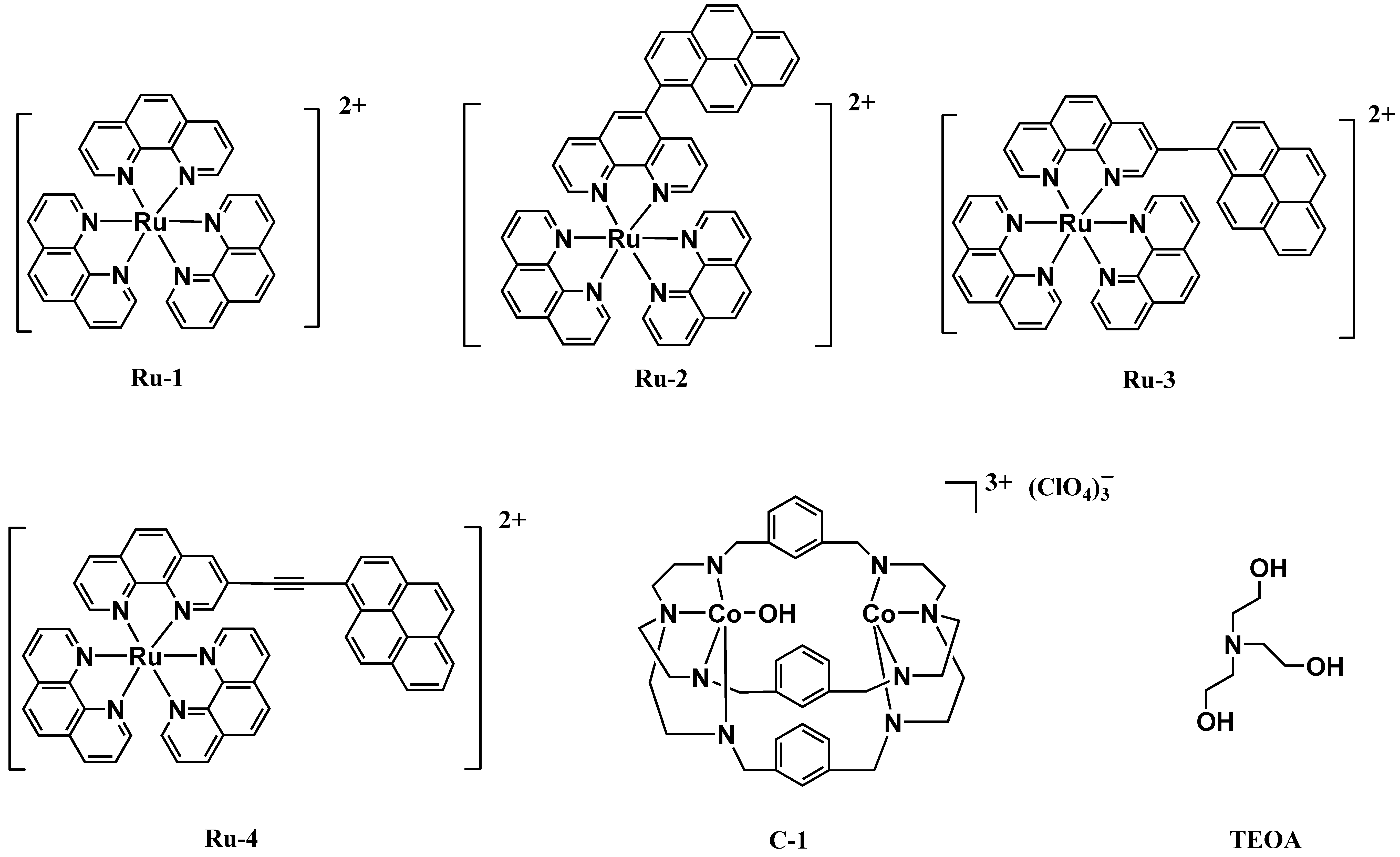


**Supplementary Scheme 2.** Molecular structure of **Ru-1** – **Ru-4**, **C-1** and **TEOA**.

**Structural characterization**


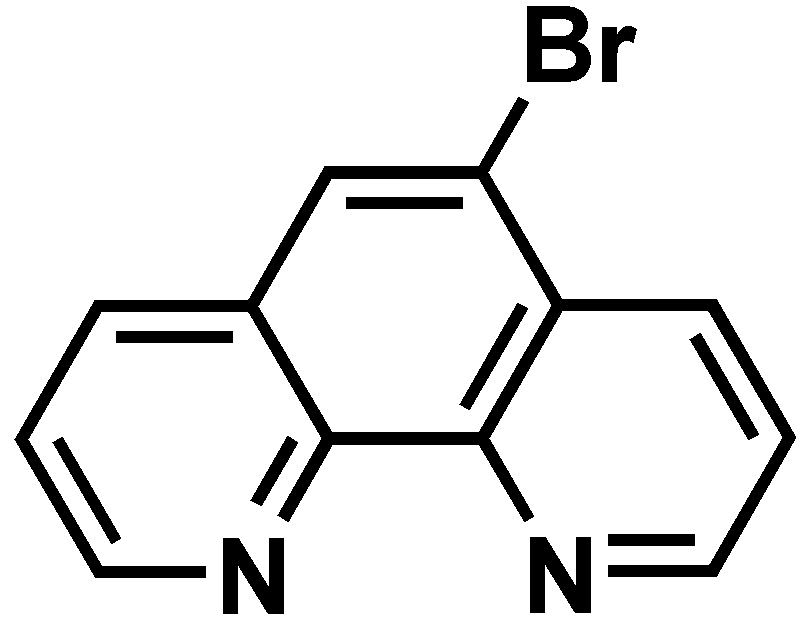


**CDCl_3_**

**Supplementary Figure 1.** ^1^H NMR spectrum of **1** (400 MHz, CDCl_3_).


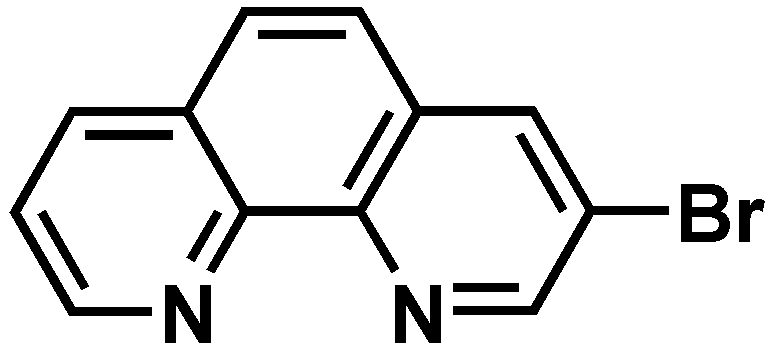


**CDCl_3_**

**Supplementary Figure 2.** ^1^H NMR spectrum of **2** (400 MHz, CDCl_3_).


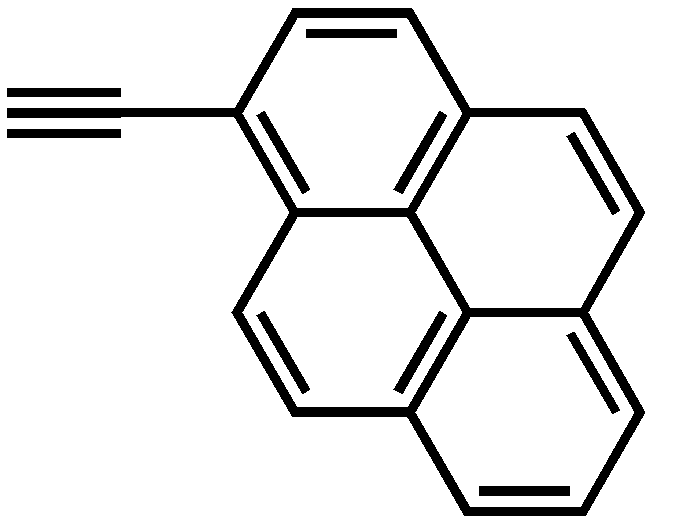


**CDCl_3_**

**Supplementary Figure 3.** ^1^H NMR spectrum of **4** (400 MHz, CDCl_3_).

**
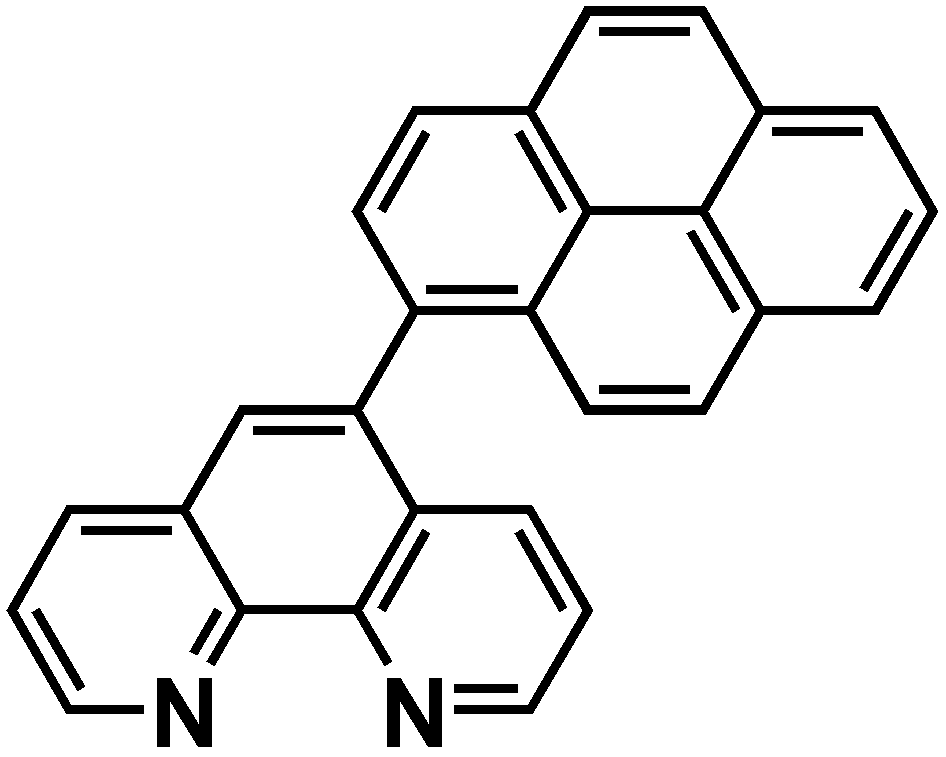
**

**CDCl_3_**

**CH_2_Cl_2_**

**n-hexane**

**CH_3_OH**

**Supplementary Figure 4.** ^1^H NMR spectrum of **L-2** (400 MHz, CDCl_3_).


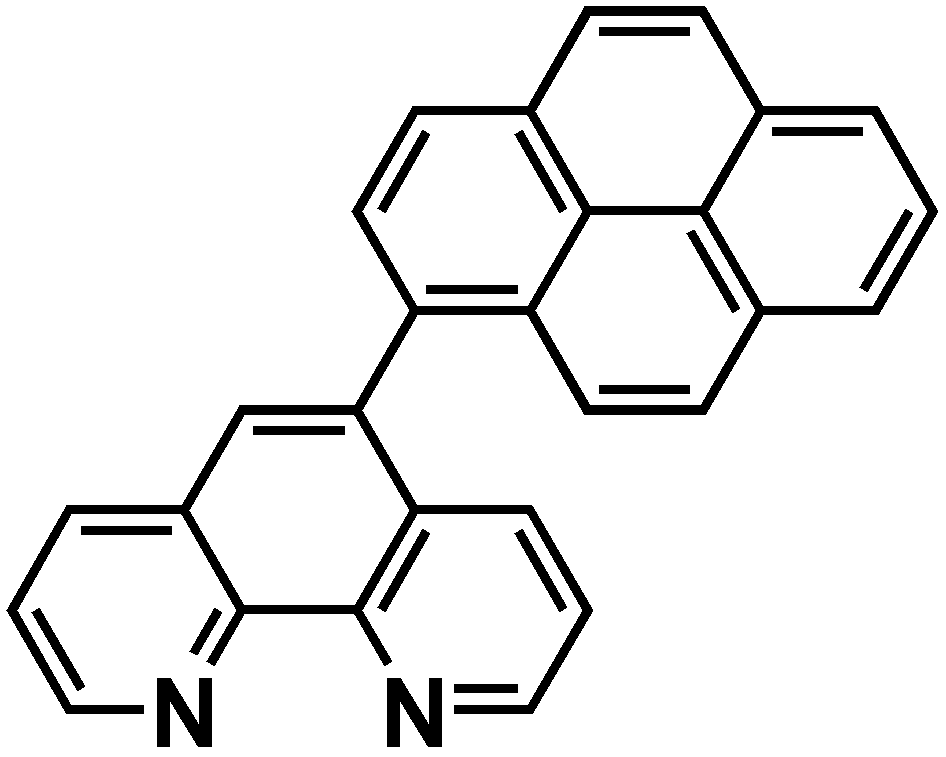

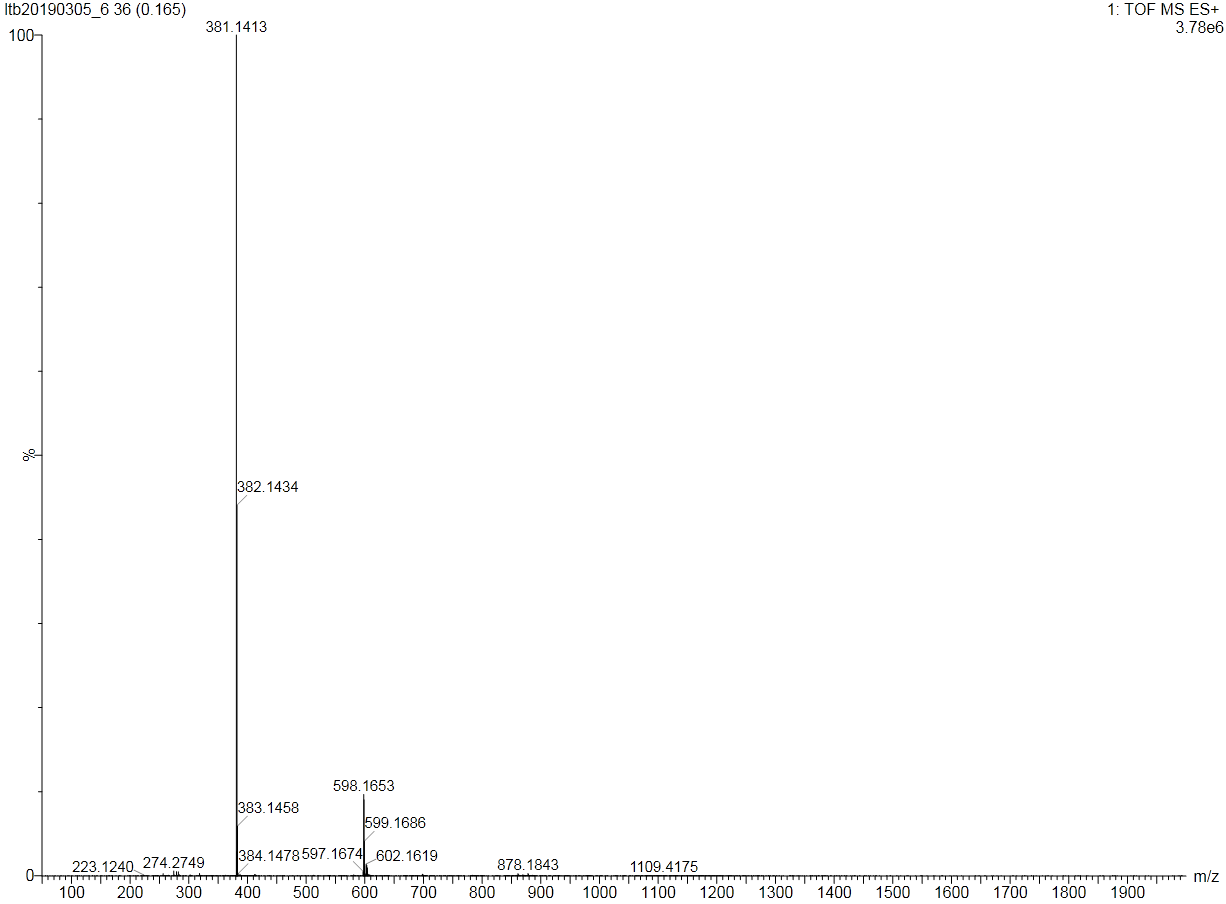


**Supplementary Figure 5.** TOF HRMS ESI of **L-2**.


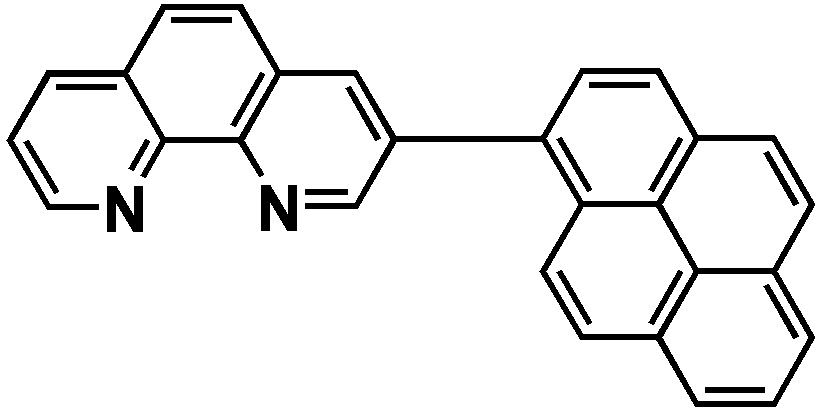


**CDCl_3_**

**CH_2_Cl_2_**

**Supplementary Figure 6.** ^1^H NMR spectrum of **L-3** (400 MHz, CDCl_3_).


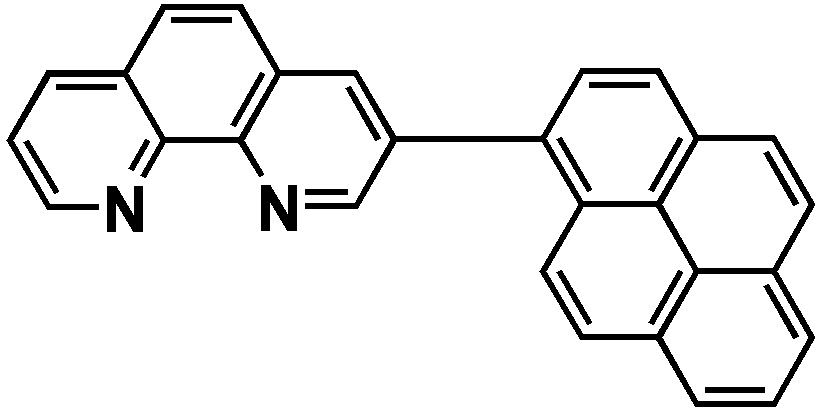

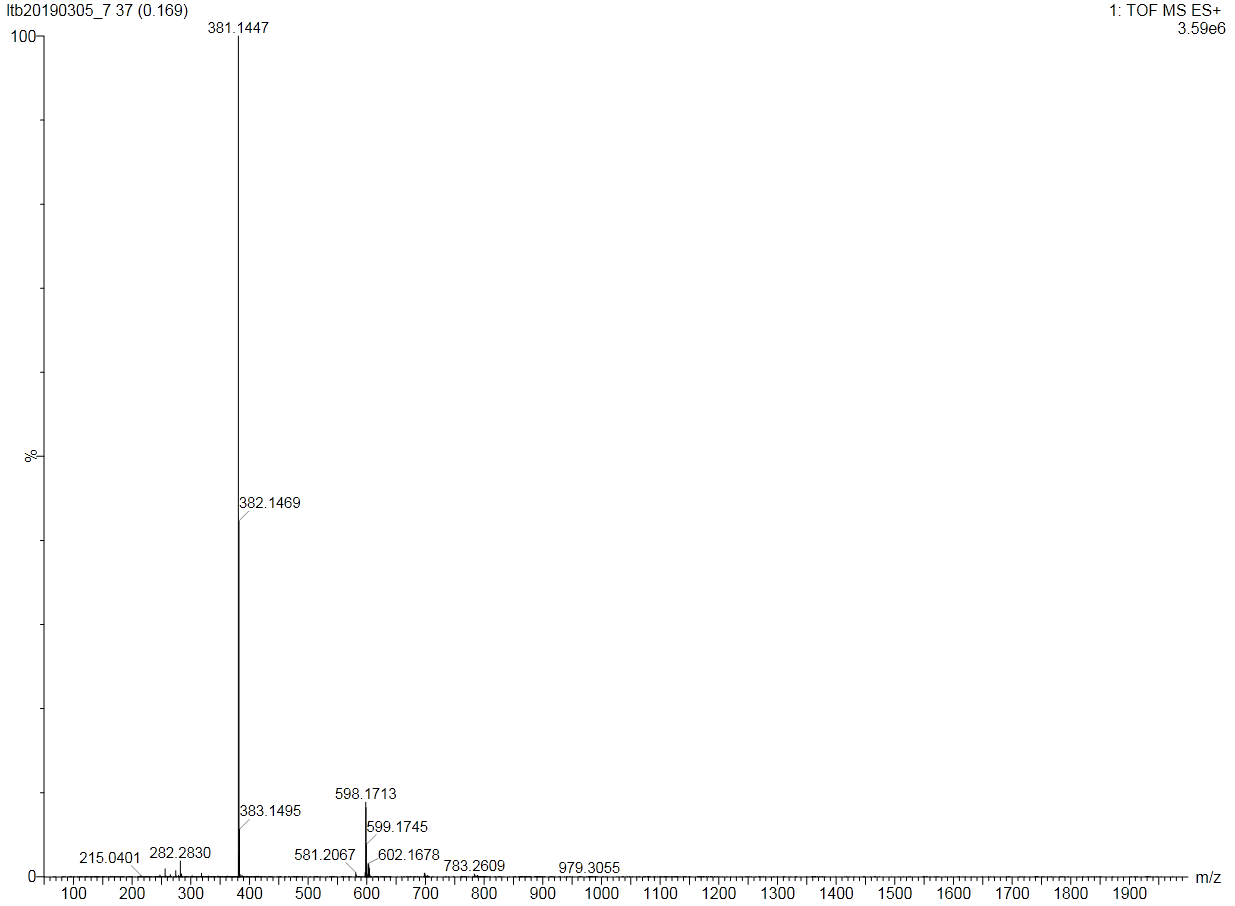


**Supplementary Figure 7.** TOF HRMS ESI of **L-3**.


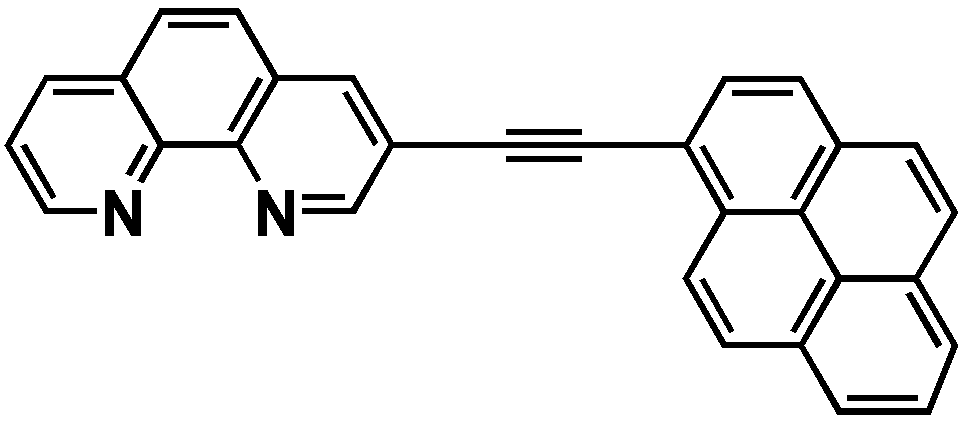


**CDCl_3_**

**n-hexane**

**CH_2_Cl_2_**

**Supplementary Figure 8.** ^1^H NMR spectrum of **L-4** (400 MHz, CDCl_3_).


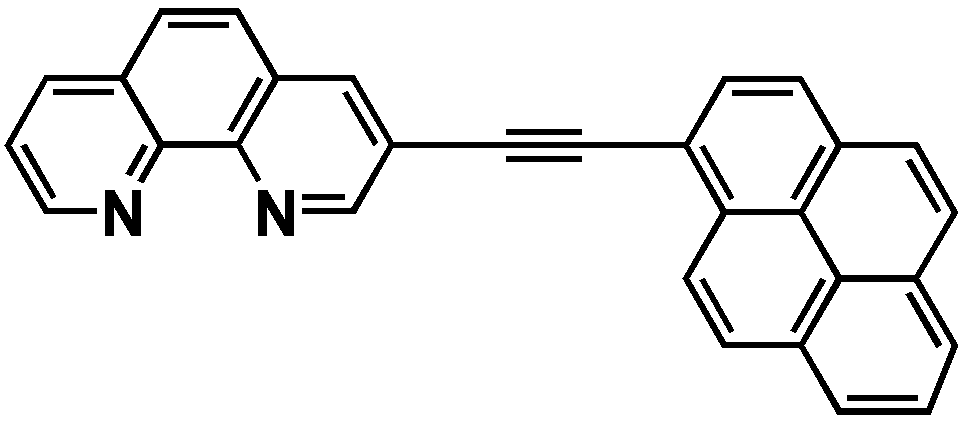

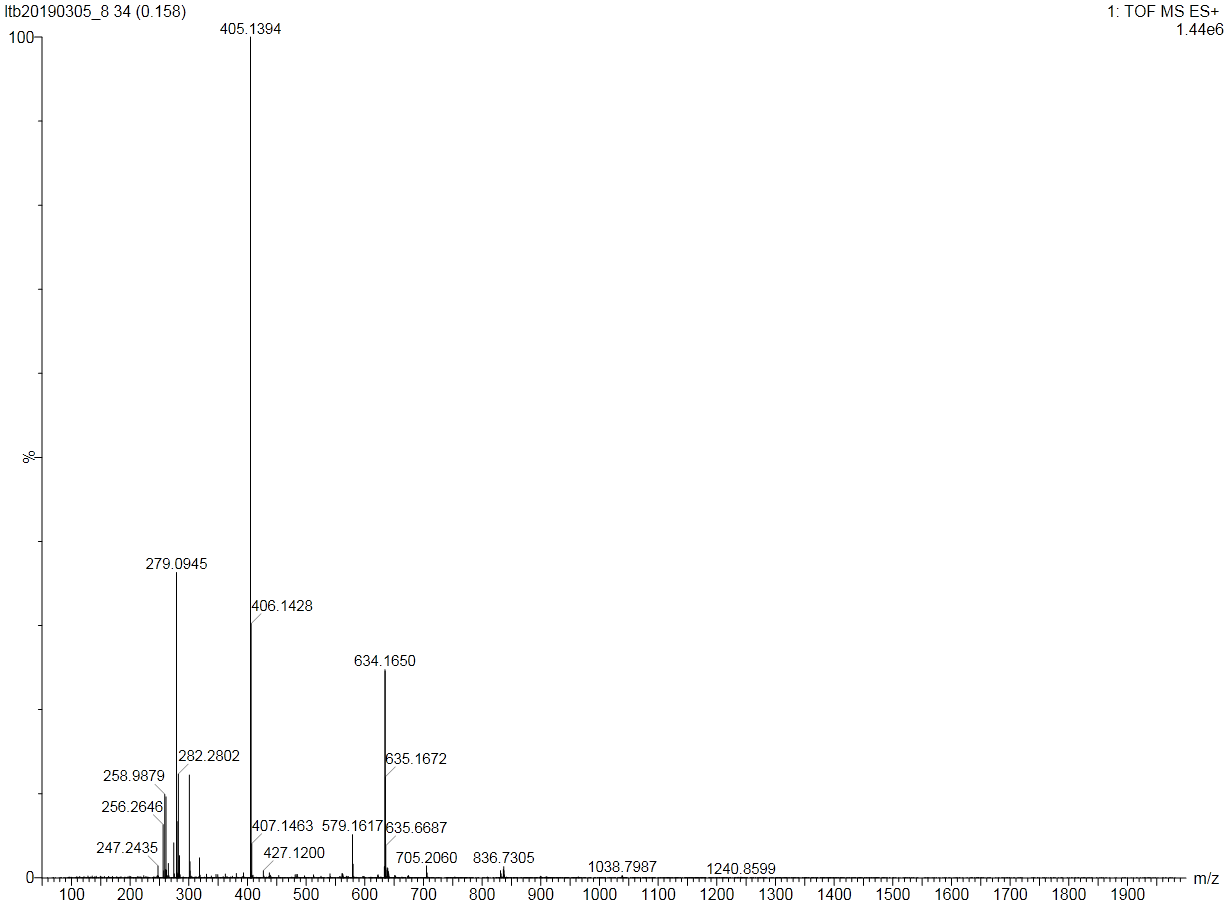


**Supplementary Figure 9.** TOF HRMS ESI of **L-4**.


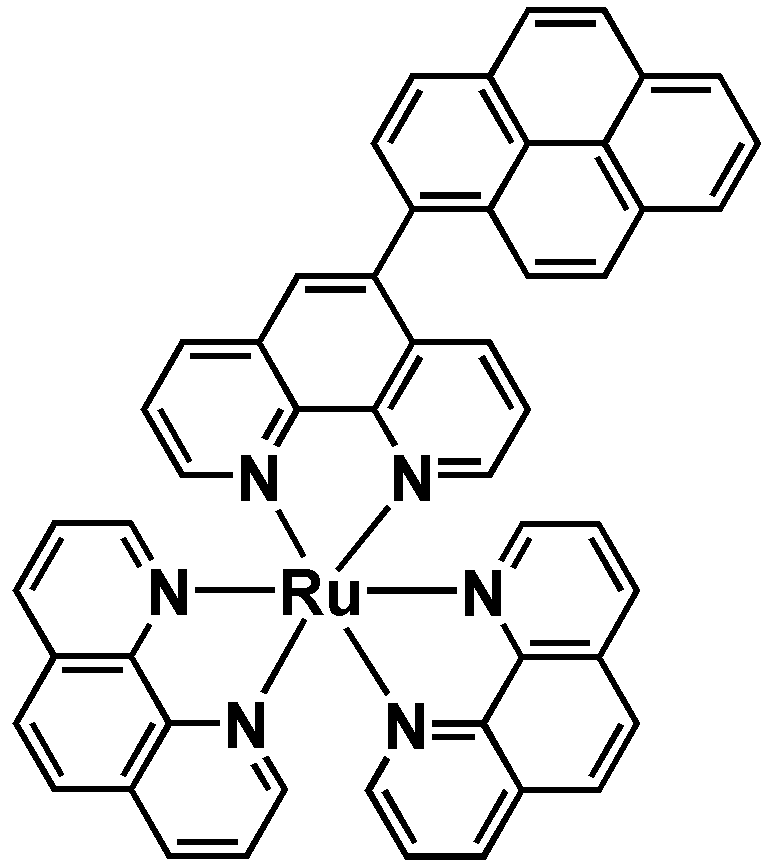


**(CD_3_)_2_CO**

**n-hexane**

**H_2_O**

**Supplementary Figure 10.** ^1^H NMR spectrum of **Ru-2** (400 MHz, *d^6^*-acetone).


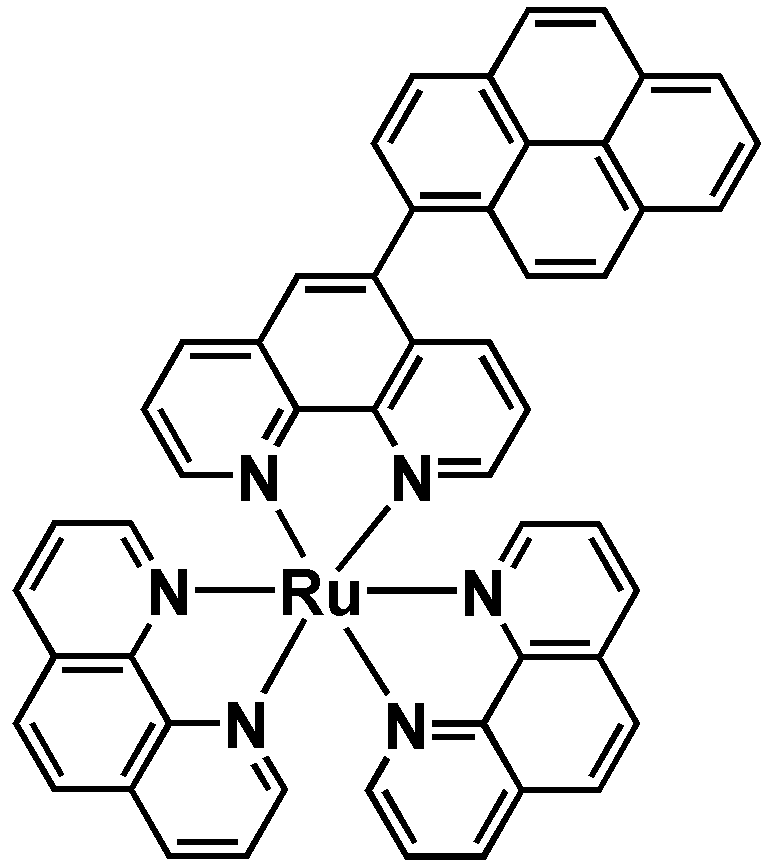


**Supplementary Figure 11.** ^13^C NMR spectrum of **Ru-2** (100 MHz, *d^6^*-acetone).


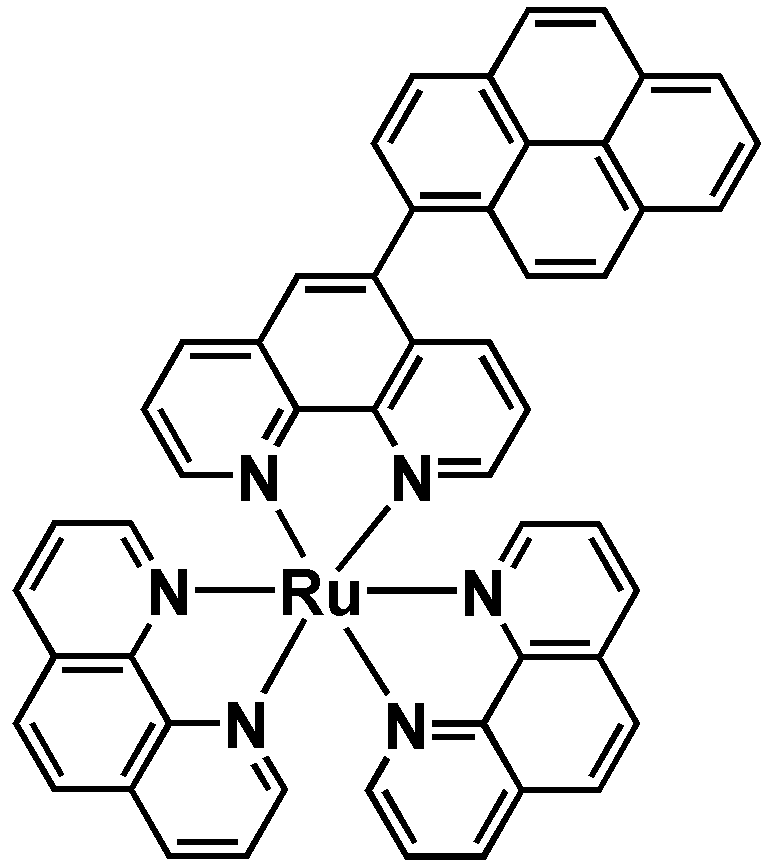

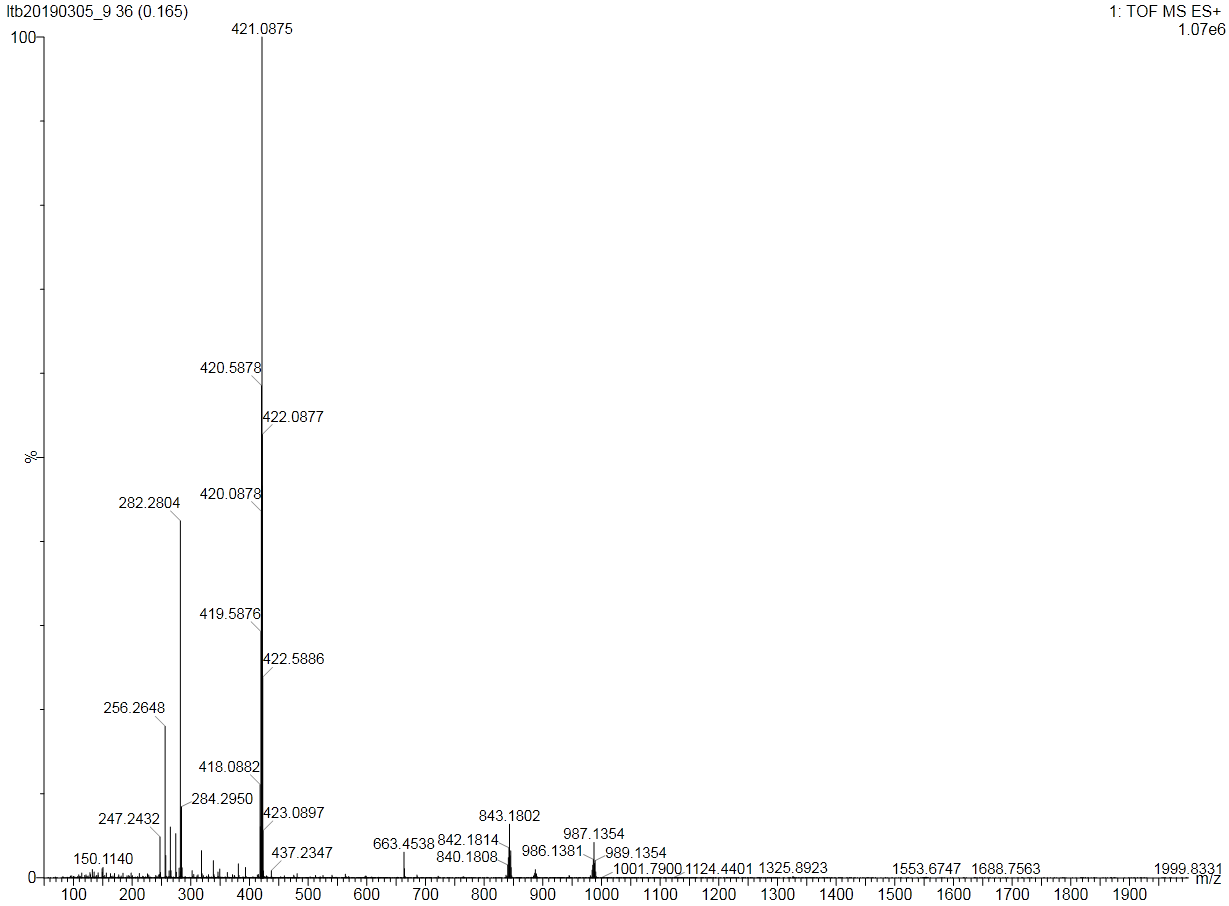


**Supplementary Figure 12.** TOF HRMS ESI of **Ru-2**.


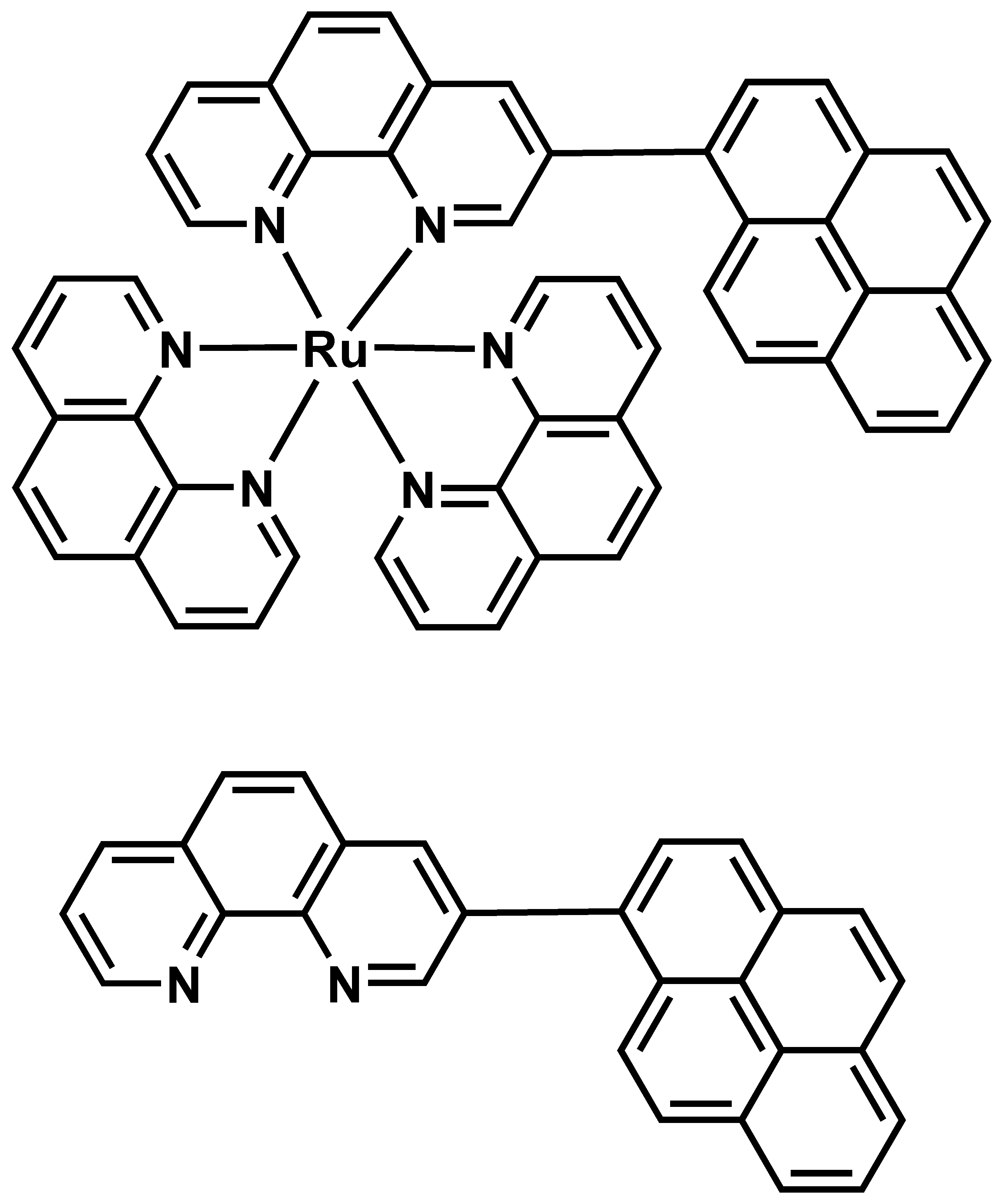


**n-hexane**

**(CD_3_)_2_SO**

**H_2_O**

**CH_2_Cl_2_**

**Supplementary Figure 13.** ^1^H NMR spectrum of **Ru-3** (400 MHz, DMSO-*d^6^*).


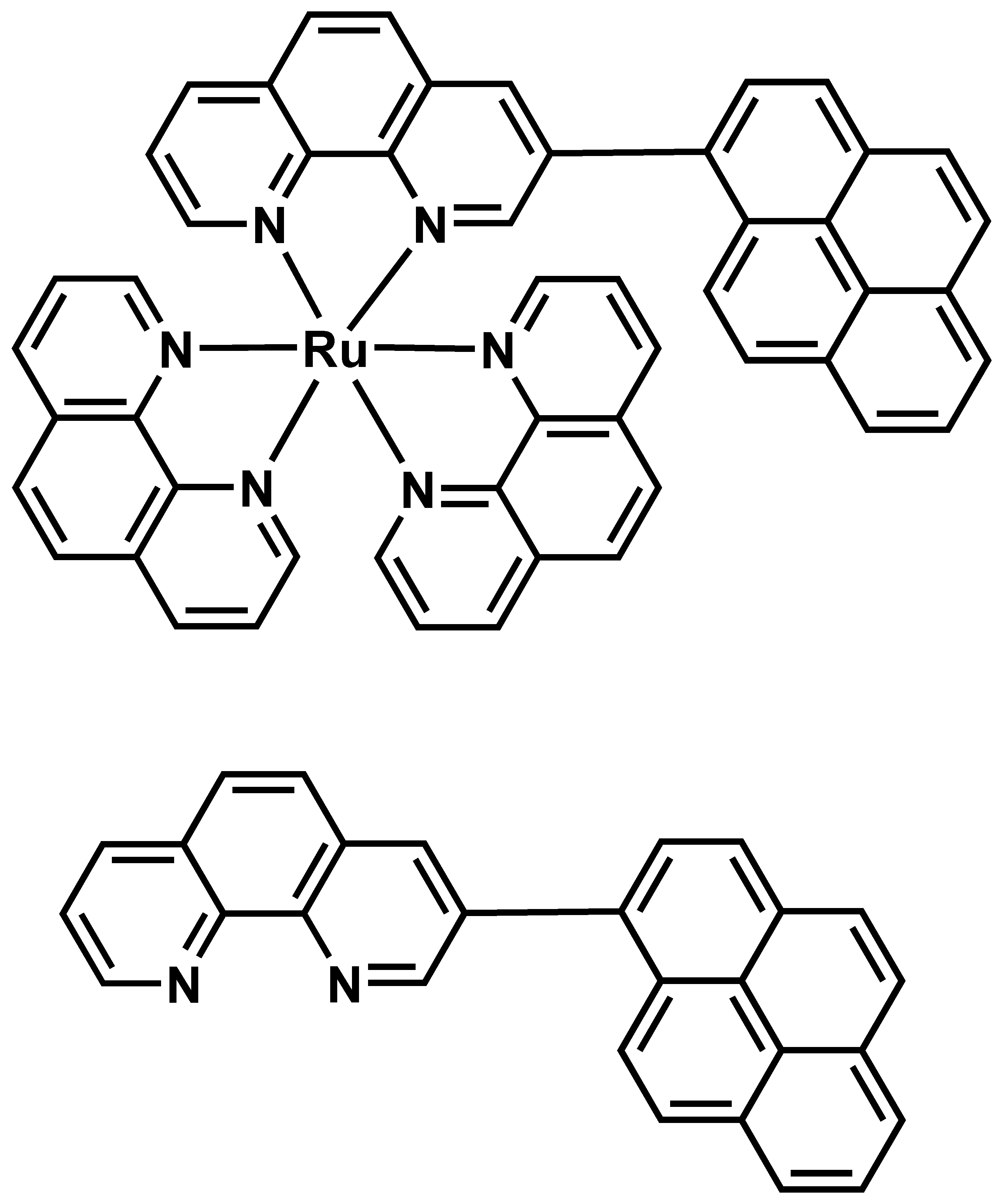


**Supplementary Figure 14.** ^13^C NMR spectrum of **Ru-3** (100 MHz, *d^6^*-acetone).


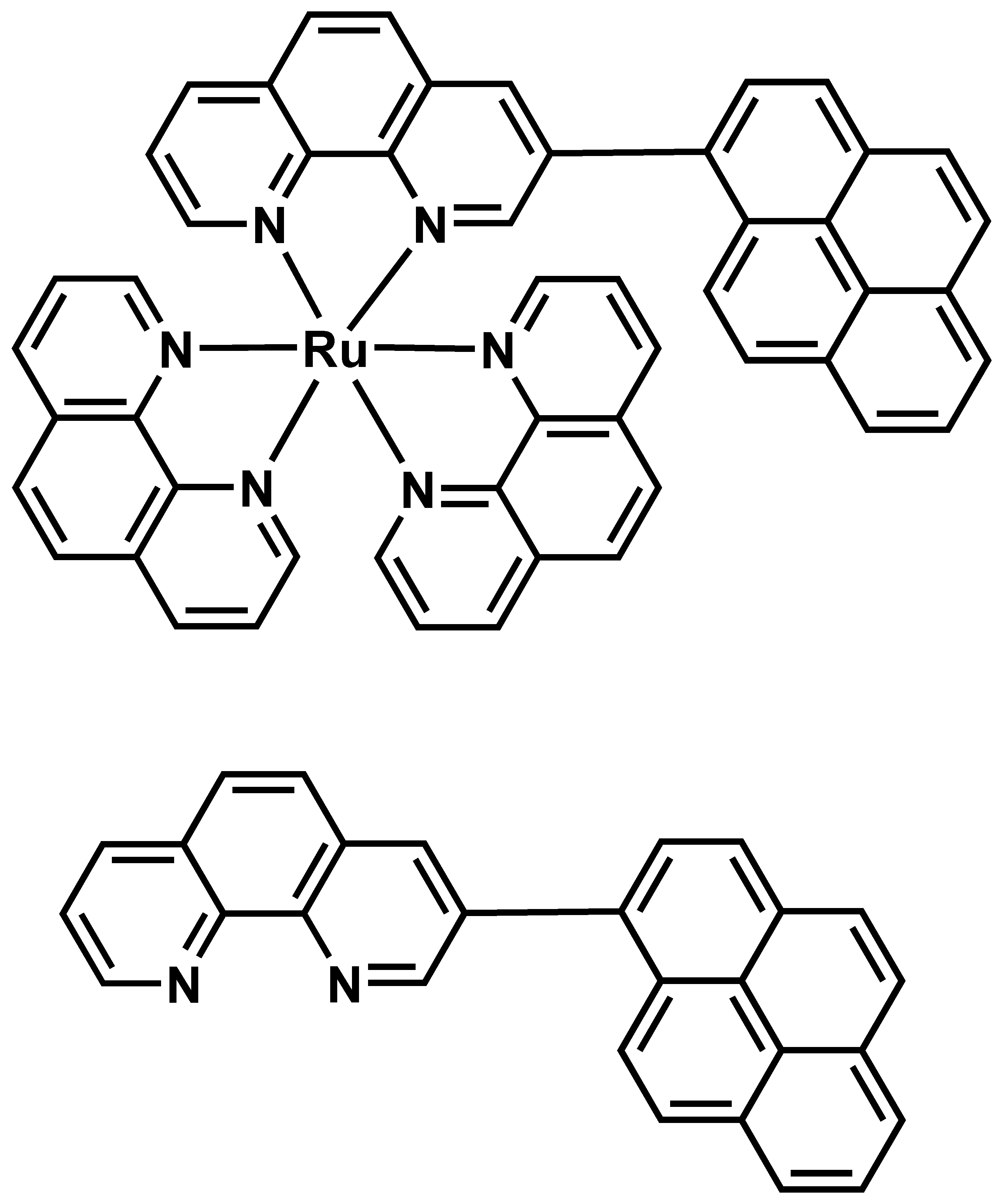

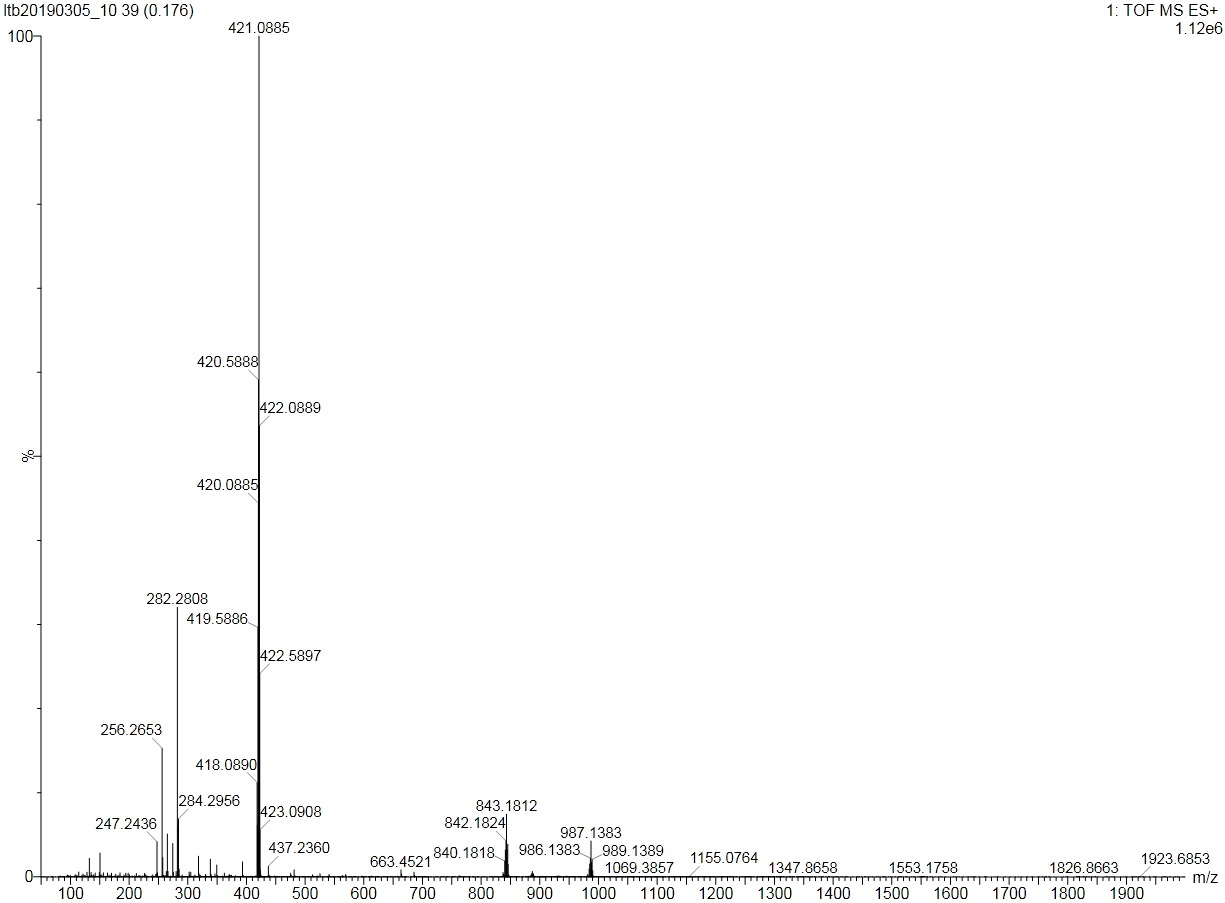


**Supplementary Figure 15.** TOF HRMS ESI of **Ru-3**.


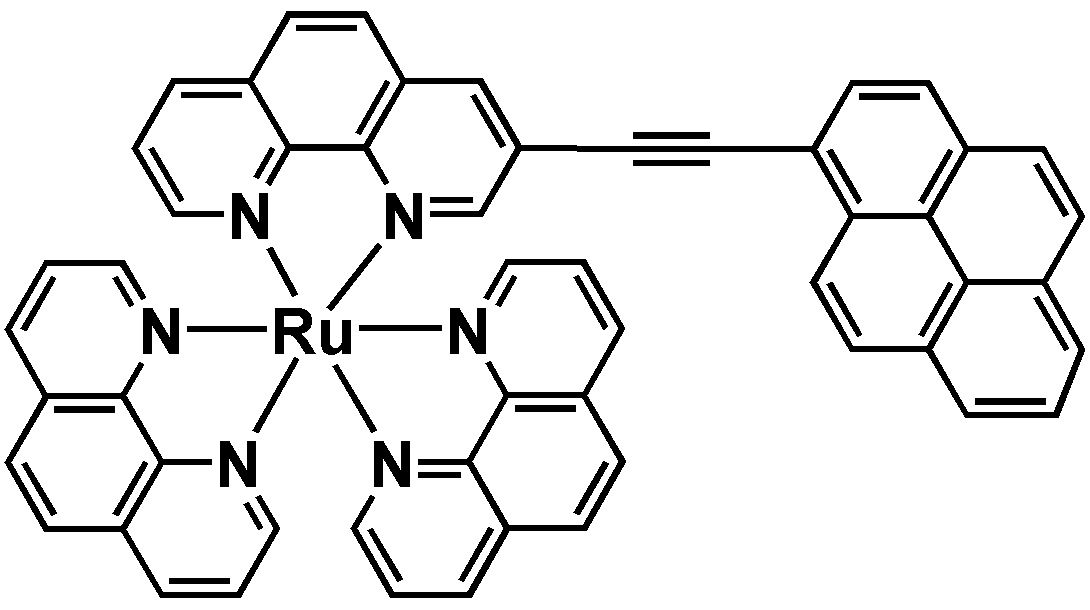


**n-hexane**

**(CD_3_)_2_SO**

**H_2_O**

**CH_2_Cl_2_**

**Supplementary Figure 16.** ^1^H NMR spectrum of **Ru-4** (400 MHz, DMSO-*d^6^*).


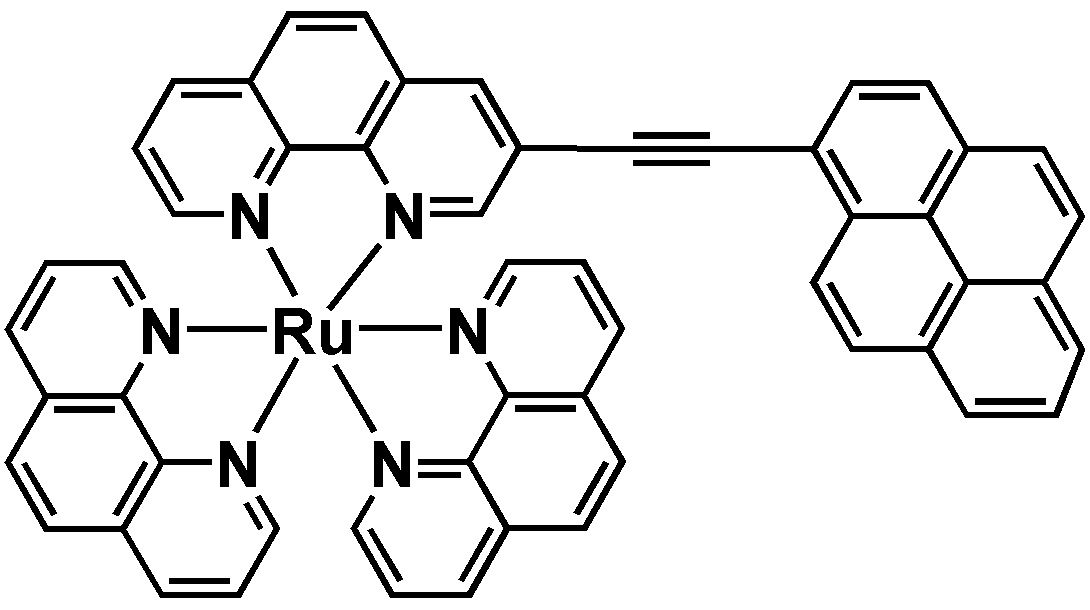


**Supplementary Figure 17.** ^13^C NMR spectrum of **Ru-4** (100 MHz, *d^6^*-acetone).


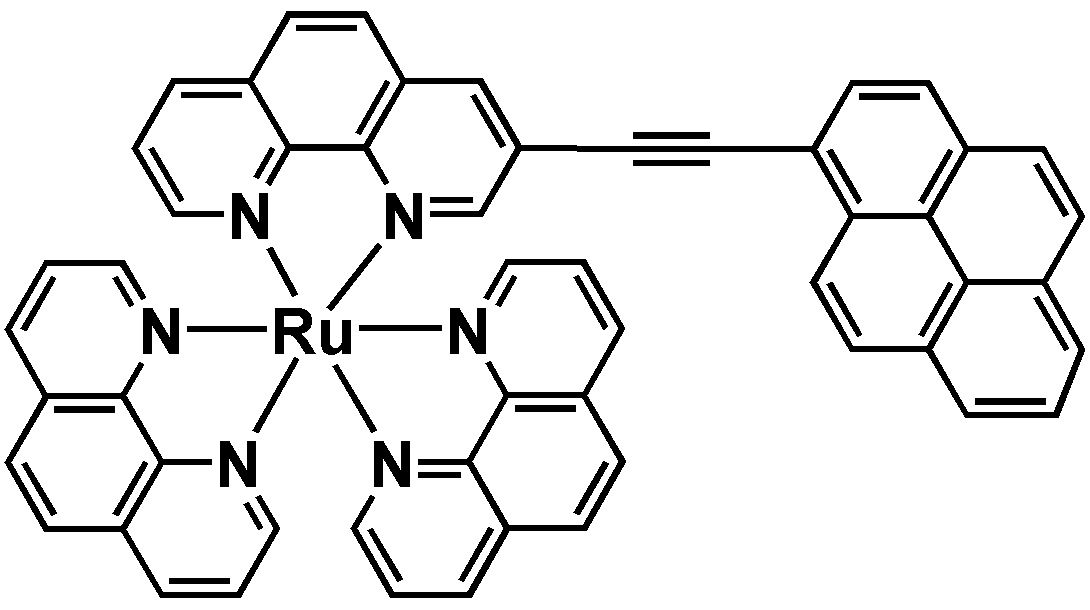

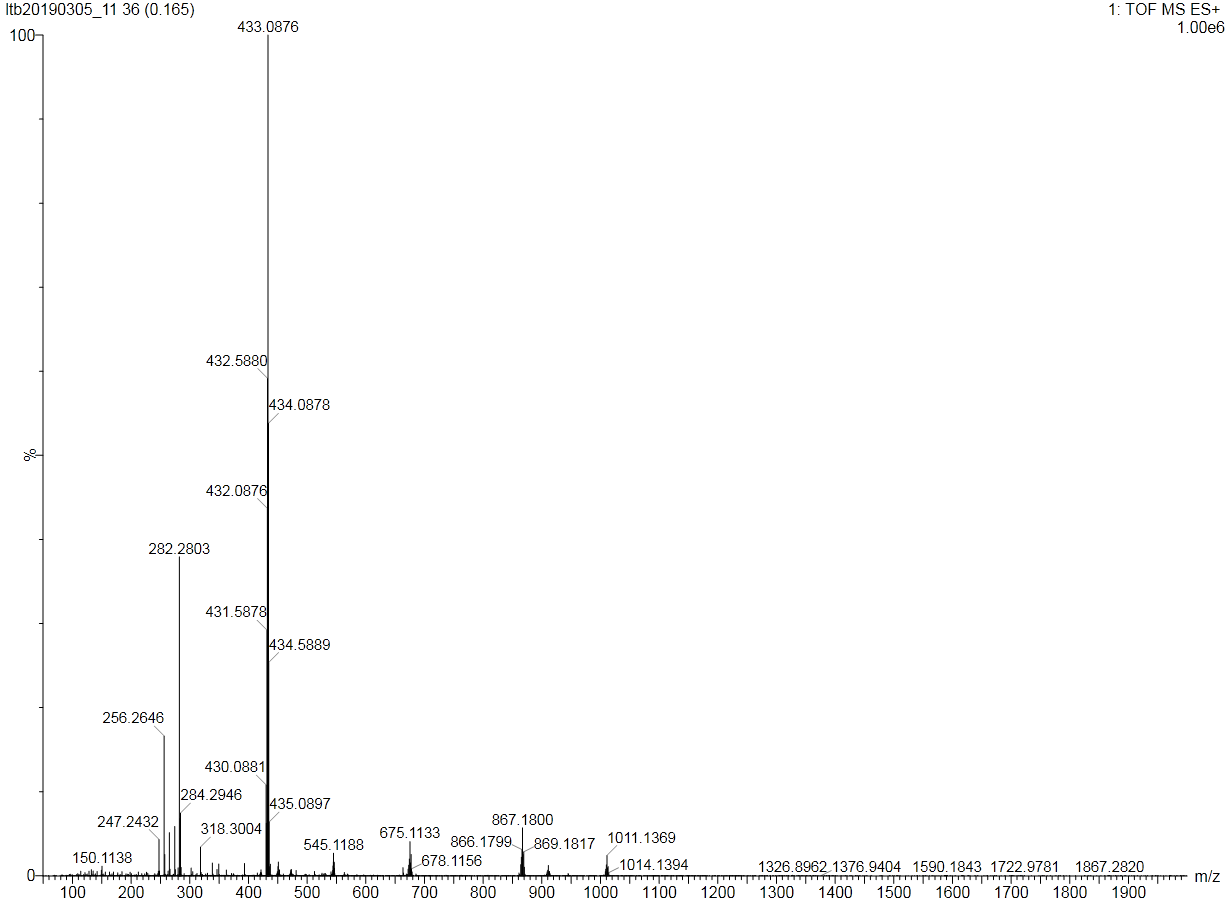


**Supplementary Figure 18.** TOF HRMS ESI of **Ru-4**.

**DFT calculations.**

**
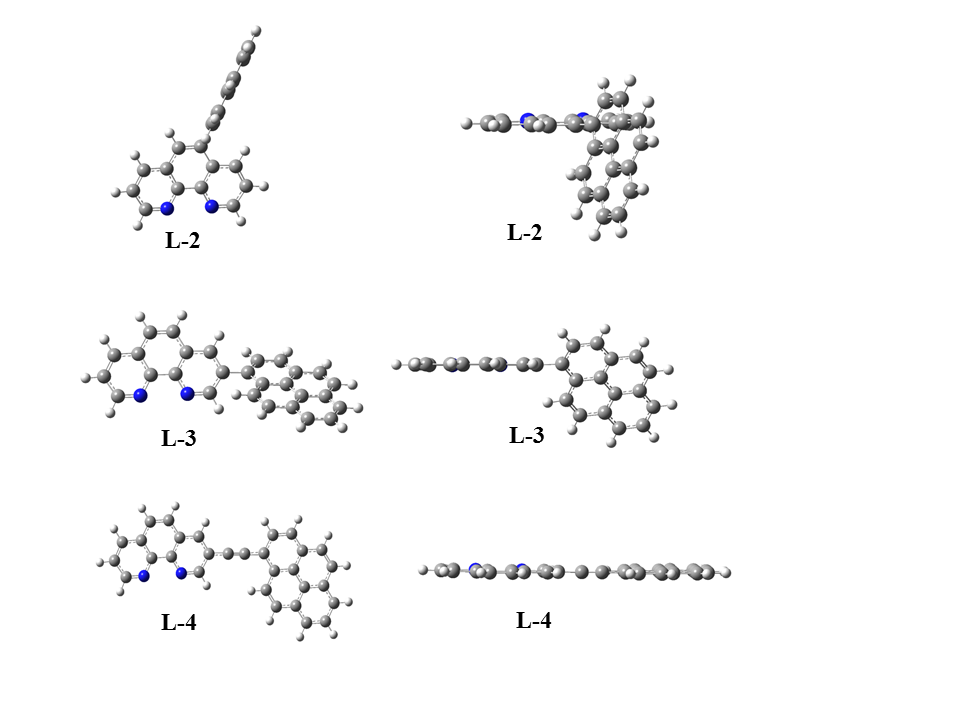
**

**Supplementary Figure 19.** The optimized structure of **L-2** – **L-4**. Calculation was performed at B3LYP/6-31G(d) level with Gaussian 09W.

Supplementary Table 1. Selected electronic excitation energies (eV) and corresponding oscillator strengths (*f*), main configurations and CI coefficients of the low-lying electronically excited states of ligands (L-2 – L-4).*^a^*

|  | Modify Dihedral *^b^* | Electronic transition | TDDFT//B3LYP/6-31G(d) | | | |
| --- | --- | --- | --- | --- | --- | --- |
|  |  |  | Energy *^c^* | *f ^d^* | Composition *^e^* | CI *^f^* |
| **L-2** | 71.5 ° | S_0_→T_1_ | 2.09 eV  594 nm | 0.0000 | H→L | 0.6588 |
| **L-3** | 55.5 ° | S_0_→T_1_ | 2.03 eV  608 nm | 0.0000 | H→L | 0.6362 |
| **L-4** | 0.0 ° | S_0_→T_1_ | 1.82 eV  682 nm | 0.0000 | H→L | 0.6523 |

*^a^*Calculated by TDDFT//B3LYP/6-31G(d). FL stands for fluorescence. *^b^*Between 1,10-Phenanthroline and pyrenyl. *^c^*Only selected low-lying excited states are presented. *^d^*Oscillator strength. *^e^*H stands for HOMO and L stands for LUMO. Only the main configurations are presented. *^f^*CI coefficients are in absolute values.

**
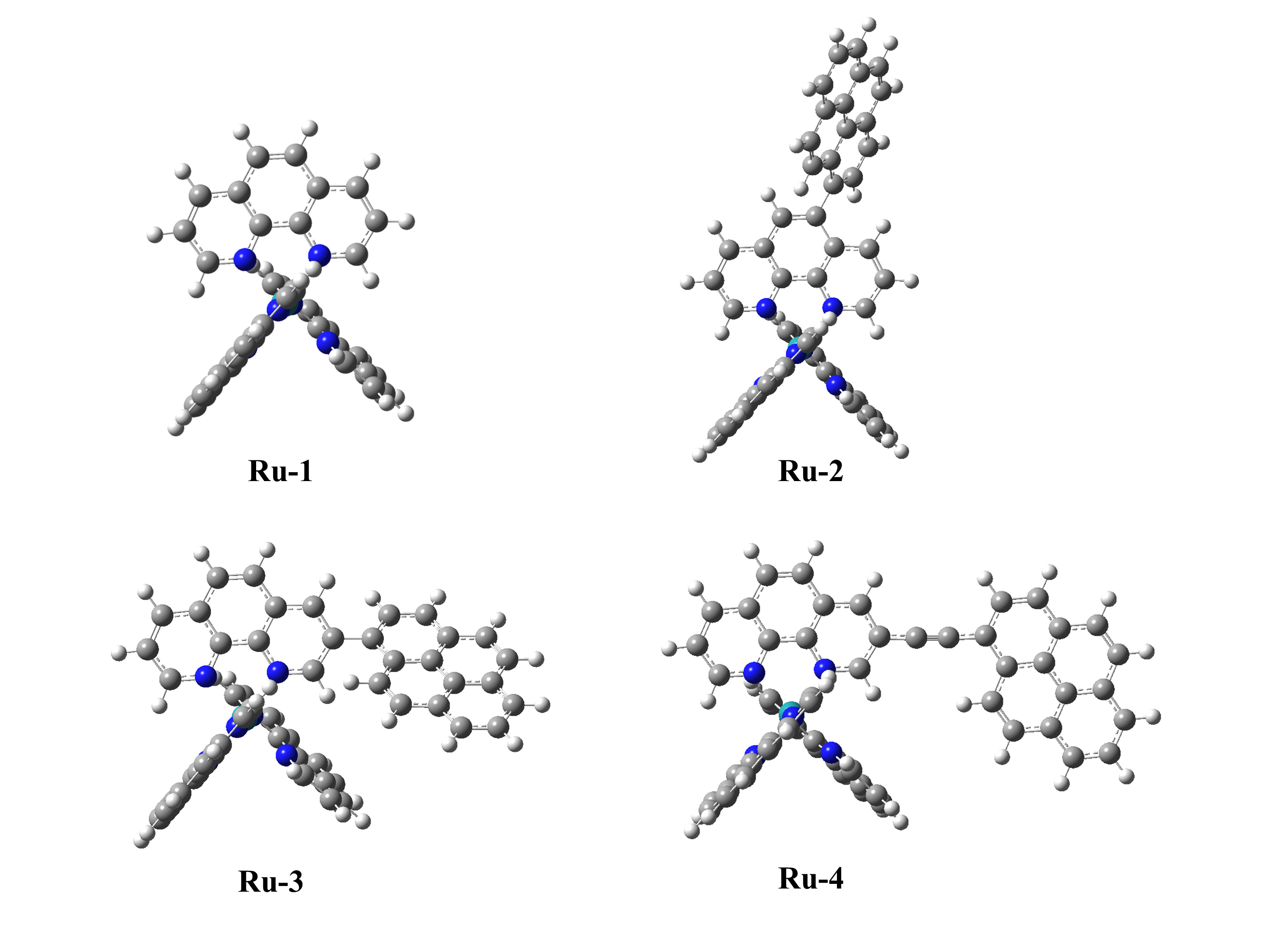
**

**Supplementary Figure 20.** The optimized structure of **Ru-1** – **Ru-4**. Calculation was performed at B3LYP/

6–31 G/genecp/LanL2DZ level with Gaussian 09 W.

**UV-vis absorption and Phosphorescence emission spectra.**

**
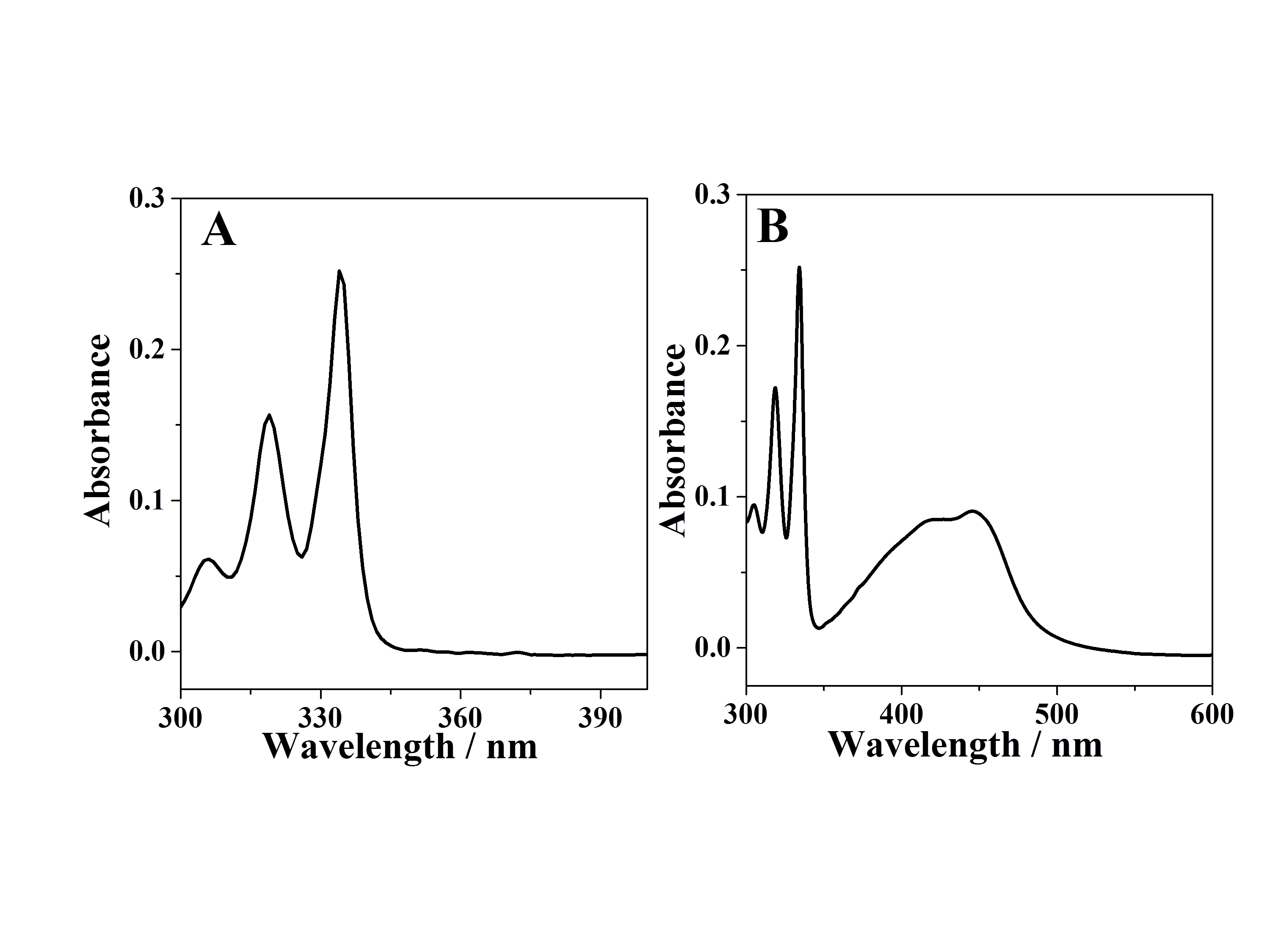
**

**Supplementary Figure 21.** UV−vis absorption spectra of (A) 5 μM pyrene, (B) 5 μM **Ru-1** in the presence of pyrene (5 μM) in CH_3_CN.

**
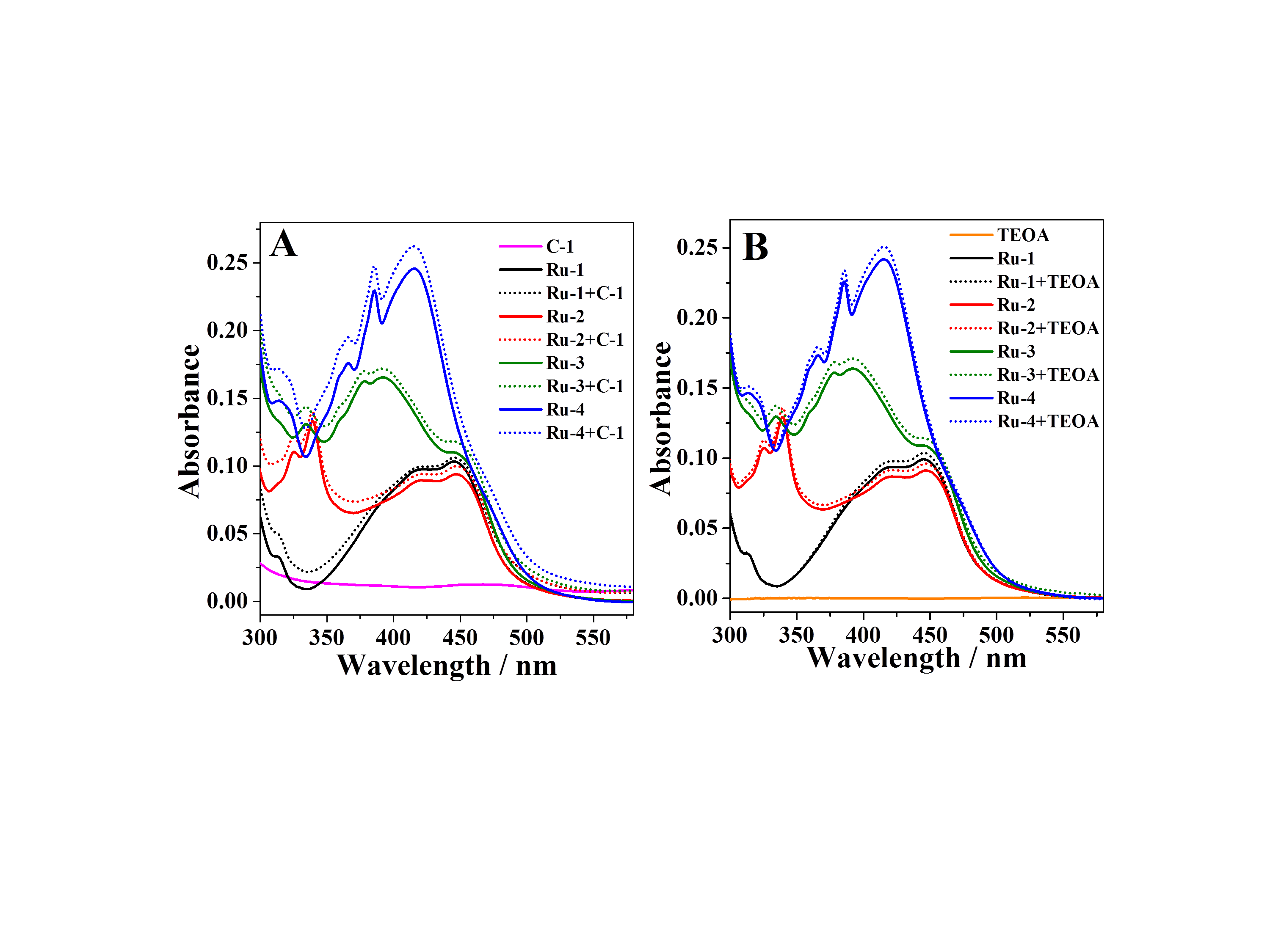
**

**Supplementary Figure 22.** UV−vis absorption spectra of a 5 μM solution of **Ru-1** – **Ru-4** in the presence of (A) **C-1** (5 μM), (B) **TEOA** (5 μM) in CH_3_CN.


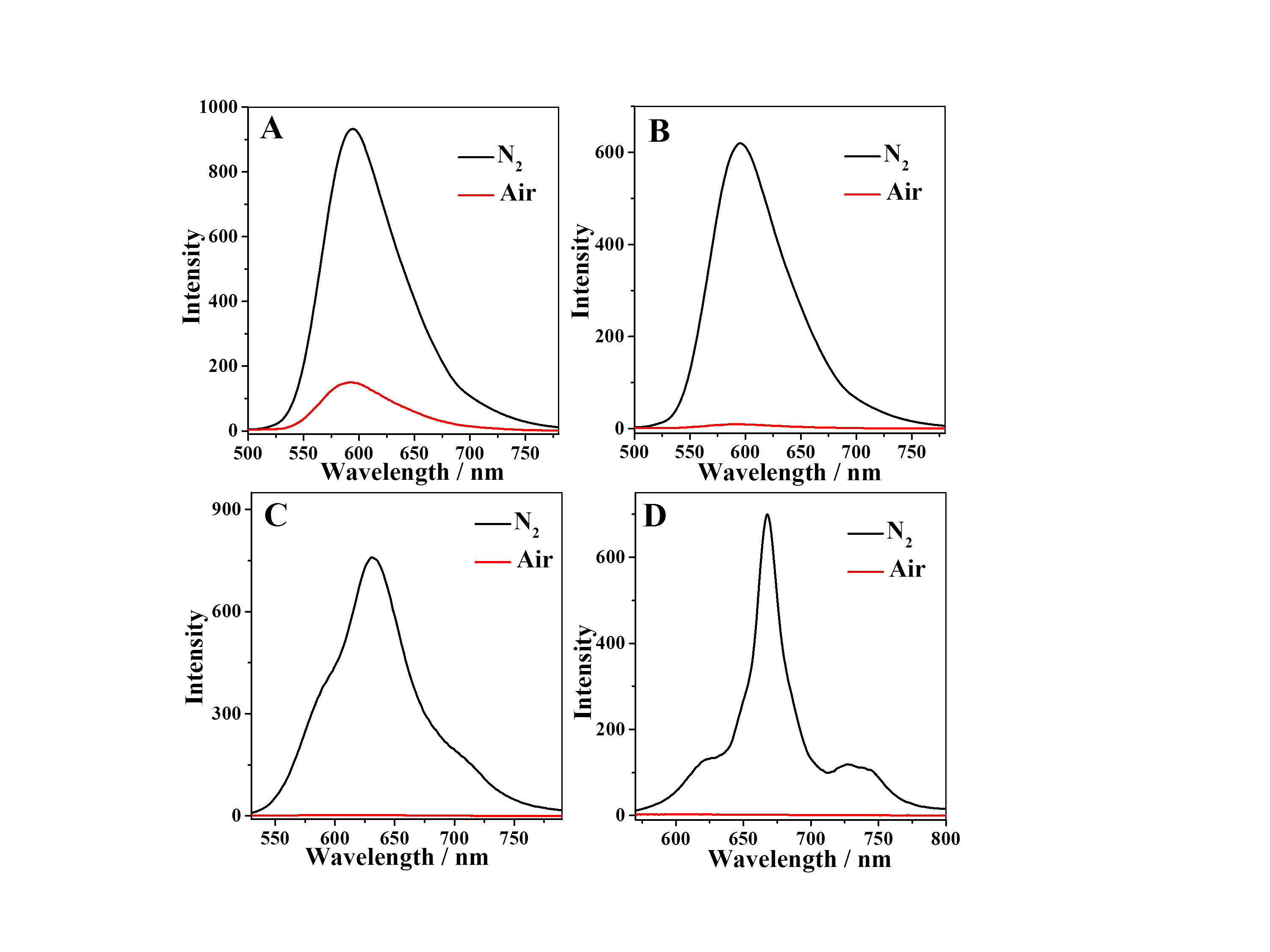


**Supplementary Figure 23.** Emission spectra of (A) **Ru-1**, (B) **Ru-2**, (C) **Ru-3**, (D) **Ru-4** in CH_3_CN under N_2_ and air atmosphere, c_PS_ = 5 μM, *λ*_ex_ = 450 nm.

**Photocatalytic CO_2_ reduction**

**Supplementary Table 2.** The results of photocatalytic reduction of CO_2_ to CO.*^a^*

| PSs | CO (μmol) | H_2_ (μmol) | Selectivity to CO | TON for CO | TOF for CO*^b^* |
| --- | --- | --- | --- | --- | --- |
| **Ru-1** | 0.33 | 0 | 100 % | 66 | 5.5 |
| **Ru-2** | 2.26 | 0 | 100 % | 452 | 37.6 |
| **Ru-3** | 5.60 | 0.29 | 95.1 % | 1120 | 93.3 |
| **Ru-4** | 0.60 | 0 | 100 % | 120 | 10 |

*^a^*20 μM PSs in the presence of **C-1** (1.0 μM) and **TEOA** (0.3 M) in 5 mL CO_2_-saturated CH_3_CN / H_2_O (*v*/*v* = 9/1) solution under irradiation with a LED light (450 nm, 100 mW·cm^-2^, irradiation area, 0.8 cm^2^). *^b^*10 h.

**Supplementary Table 3.** The results of photocatalytic reduction of CO_2_ to CO.*^a^*

| PSs | CO (μmol) | H_2_ (μmol) | TON for CO | References |
| --- | --- | --- | --- | --- |
| **Ru-1** | 2.11 | 0.046 | 16896 | Angew. Chem. Int. Ed. 2017, 56, 738 |
| **Ru-3** | 8.31 | 0.81 | 66480 | This work |

*^a^*400 μM PSs in the presence of **C-1** (0.025 μM) and **TEOA** (0.3 M) in 5 mL CO_2_-saturated CH_3_CN / H_2_O (*v*/*v* = 9/1) solution under irradiation with a LED light (450 nm, 100 mW·cm^-2^, irradiation area, 0.8 cm^2^).

**Supplementary Table 4. The results of control experiments for photocatalytic reduction of CO_2_ to CO.**

| Entry*^a^* | CO (μmol) | H_2_ (μmol) | TON |
| --- | --- | --- | --- |
| 1 | 0 | 0 | 0 |
| 2 | 0 | trace | 0 |
| 3 | 0 | 0 | 0 |
| 4 | 0 | 0 | 0 |
| 5 | 0 | 0 | 0 |
| 6 | 0 | 0.26 | 0 |

*^a^*Entry 1: without PS; Entry 2: without **C-1**; Entry 3: without **TEOA**; Entry 4: CH_3_CN as solvent; Entry 5: without light; Entry 6: Ar. Reaction conditions: **Ru-3** (20 μM), **C-1** (1 μM), **TEOA** (0.3 M), in 5 mL CO_2_-saturated CH_3_CN / H_2_O (*v*/*v* = 9/1) solution, LED light (450 nm, 100 mW•cm^-2^, irradiation area 0.8 cm^2^), irradiation time: 18 h.


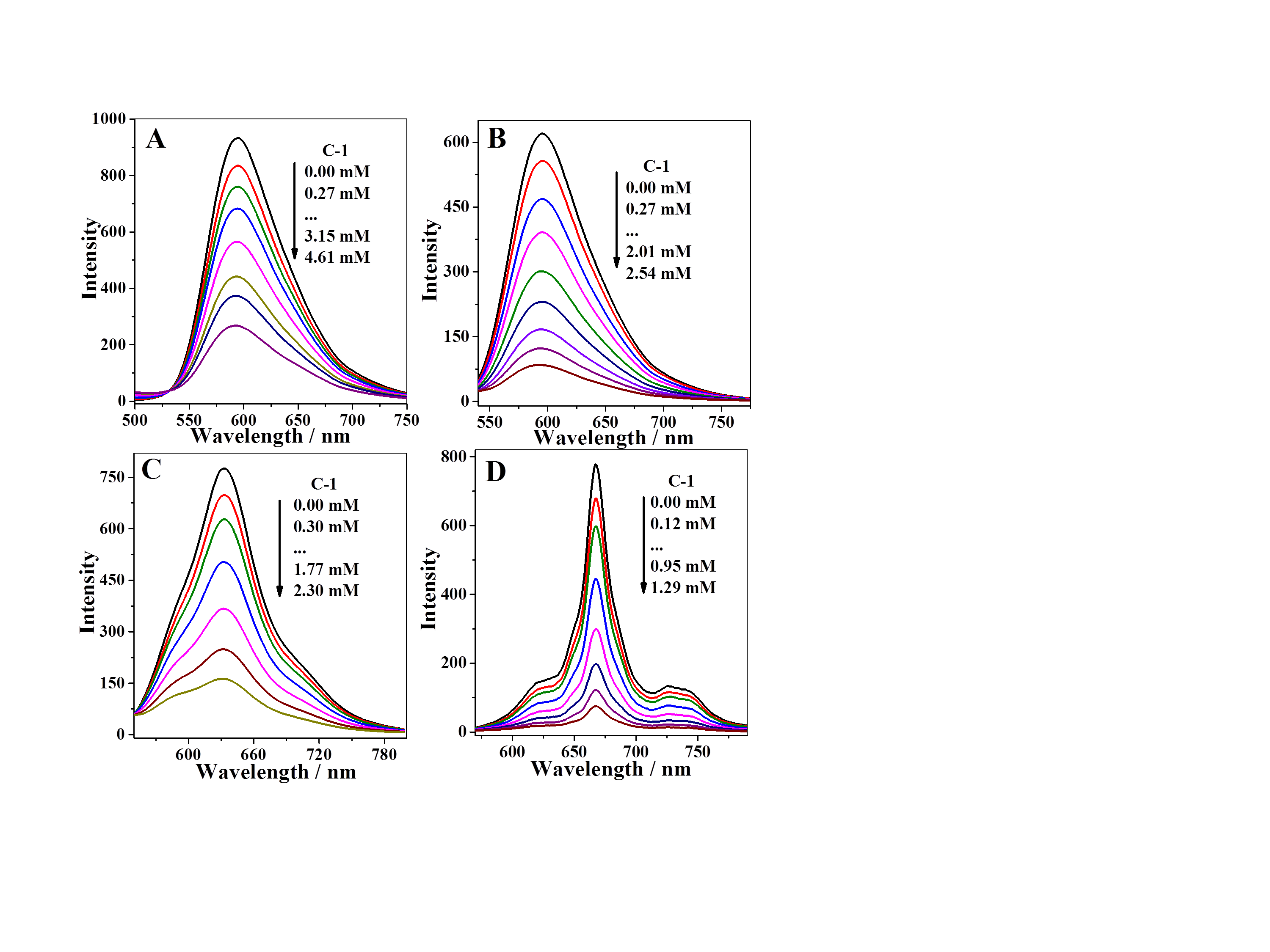


**Supplementary Figure 24**. Phosphorescence quenching of (A) **Ru-1**, (B) **Ru-2**, (C) **Ru-3**, (D) **Ru-4** with **C-1** as the quencher in acetonitrile. *λ*_ex_ = 450 nm, c_PS_ = 5.0 μM.


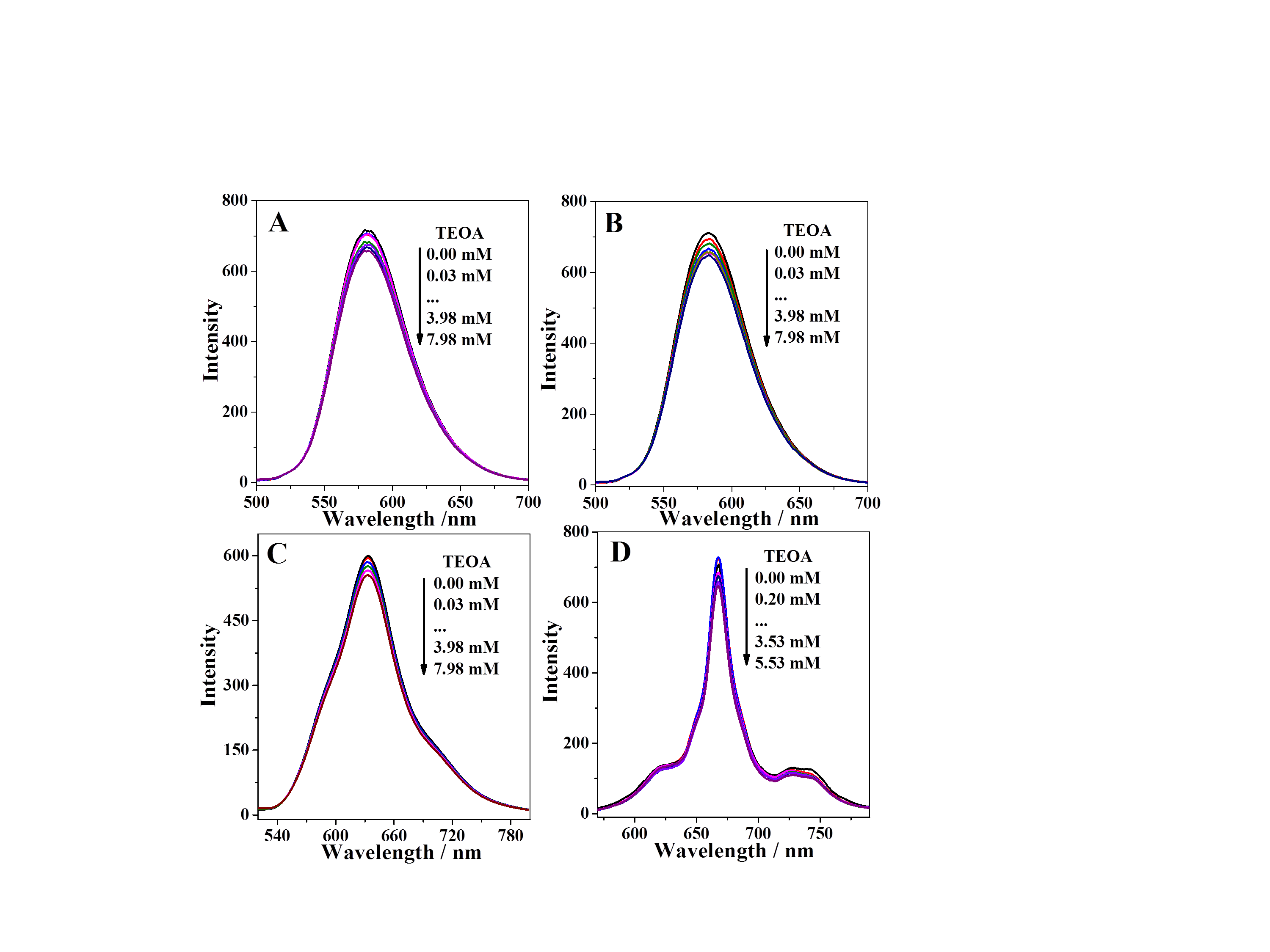


**Supplementary Figure 25**. Phosphorescence quenching of (A) **Ru-1**, (B) **Ru-2**, (C) **Ru-3**, (D) **Ru-4** with **TEOA** as the quencher in acetonitrile. *λ*_ex_ = 450 nm, c_PS_ = 5.0 μM.


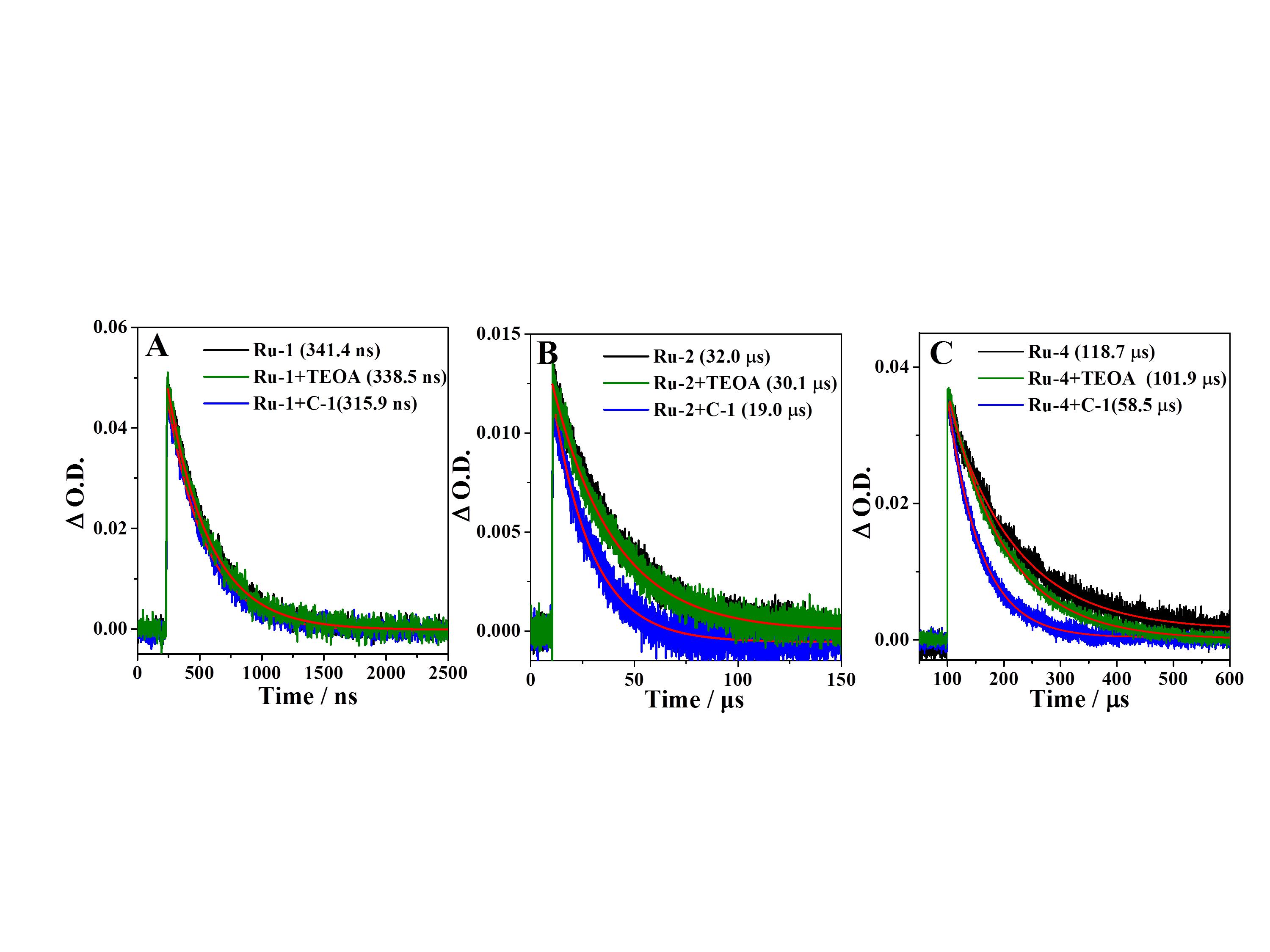


**Supplementary Figure 26.** Nanosecond transient absorption spectra of kinetic decay trace of (A) **Ru-1** (black), **Ru-1** with 0.4 mM TEOA (green), **Ru-1** with 0.5 mM **C-1** (blue); (B) **Ru-2** (black), **Ru-2** with 0.4 mM TEOA (green), **Ru-2** with 0.5 mM **C-1** (blue); (C) **Ru-4** (black), **Ru-4** with 0.4 mM TEOA (green), **Ru-4** with 0.5 mM **C-1** (blue). Spectra were followed at 450 nm in acetonitrile after pulsed excitation at 355 nm (**Ru-2** and **Ru-3**) and at 450 nm (**Ru-1** and **Ru-4**) under N_2_.

**Cyclic voltammograms**

**Supplementary Table 5.** Redox potentials of **Ru-1** – **Ru-4** PSs and **C-1**. Ferrocene (Fc) was used as an internal reference (E_1/2_ = + 0.40 V (Fc^+^/Fc) vs. SCE). A glassy carbon was used as working electrode, a platinum counter electrode and a Ag/AgNO_3_ reference electrode.

|  | **E (Ru^2+^/Ru^3+^)** | **E (L/L^-^)** | **E_0,0_** | ***Ox** | ***Red** |
| --- | --- | --- | --- | --- | --- |
| **Ru-1** | 1.29 | -1.31, -1.47 | 2.33 | -1.04 | 1.02 |
| **Ru-2** | 1.36 | -1.27, -1.43 | 2.32 | -0.96 | 1.05 |
| **Ru-3** | 1.36 | -1.27, -1.45 | 2.28 | -0.92 | 1.01 |
| **Ru-4** | 1.40 | -0.71, -1.07, -1.42 | 2.10 | -0.70 | 1.39 |
| **C-1** | - | -1.33, -1.51 | - | - | - |
| **TEOA** | 1.01 | - | - | - | - |

*Ox = E _(Ru_^2+^_/Ru_^3+^) – E_00_ (where E _(Ru_^2+^_/Ru_^3+^) is the ground state oxidation potential)

*Red = E _(L/L_^-^) + E_00_ (where E _(L/L_^-^) is the ground state reduction potential)

The value of E_0,0_ estimated from the onset (5% relative intensity) of the room-temperature emission spectrum.
